# Supplementary material for: Brachiopod genome unveils the evolution of BMP signalling in bilaterian body patterning
Source: Nat Commun. 2026 Mar 12;17:3856. doi: 10.1038/s41467-026-70403-5 (PMC13121700; doi:10.1038/s41467-026-70403-5)
Supplement: Supplementary file 1 — Supplementary Information [file 41467_2026_70403_MOESM1_ESM.pdf]

Supplementary Information for

Brachiopod genome unveils the evolution of BMP signalling in bilaterian body patterning

Thomas D. Lewin, Tosuke Sakagami, Keisuke Shimizu, Li-Jung Kao, Yi-Ling Chiu, Isabel Jiah-Yih Liao, Mu-En Chen, Kanako Hisata, Kazuyoshi Endo, Noriyuki Satoh, Peter W. H. Holland, Yue Him Wong*, Yi-Jyun Luo*

*Corresponding authors: Yue Him Wong (timwong@szu.edu.cn), Yi-Jyun Luo (yjluo@as.edu.tw)

This PDF file includes:

Supplementary Figs. 1 to 32

Legends for Supplementary Data 1 to 40

Other supporting materials for this manuscript include:

Supplementary Data 1 to 40

Supplementary Figures 1 to 32

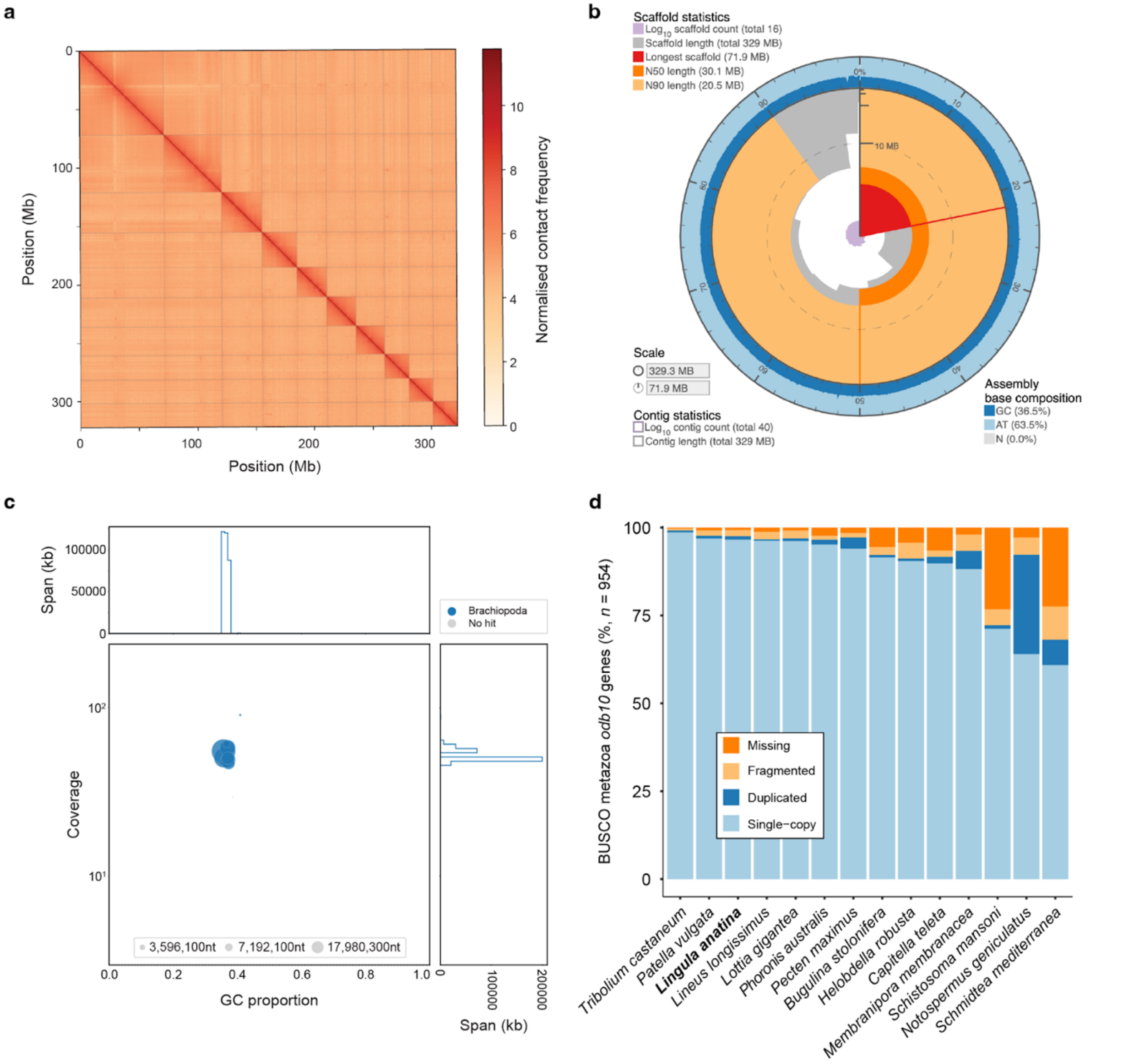

Supplementary Fig. 1 | A chromosome-level assembly for the brachiopod *Lingula anatina*. a, Hi-C contact map of the *L. anatina* genome assembly. Axes are sorted by chromosome size with highest at the top left. Colours represent intensity of interaction: darker colour = stronger interaction. **b**, Snail plot showing key statistics for *L. anatina* genome assembly. The 329.3 Mb assembly is divided into 16 scaffolds, 10 of which are chromosome-scale. The longest scaffold (red) is 71.9 Mb, the scaffold N50 (bright orange) is 30.1 Mb and the N90 (pale orange) is 20.5 Mb. The genome has a 36.5% GC content. **c**, Blob plot for the final *L. anatina* assembly showing GC content, coverage, scaffold length and blast hits. **d**, BUSCO metazoa *odb10* (*n* = 954) results for selected spiralian genomes. Statistics for *L. anatina*: Complete: 97.5% [Single-copy: 96.6%, Duplicated: 0.9%], Fragmented: 1.8%, Missing: 0.7%.

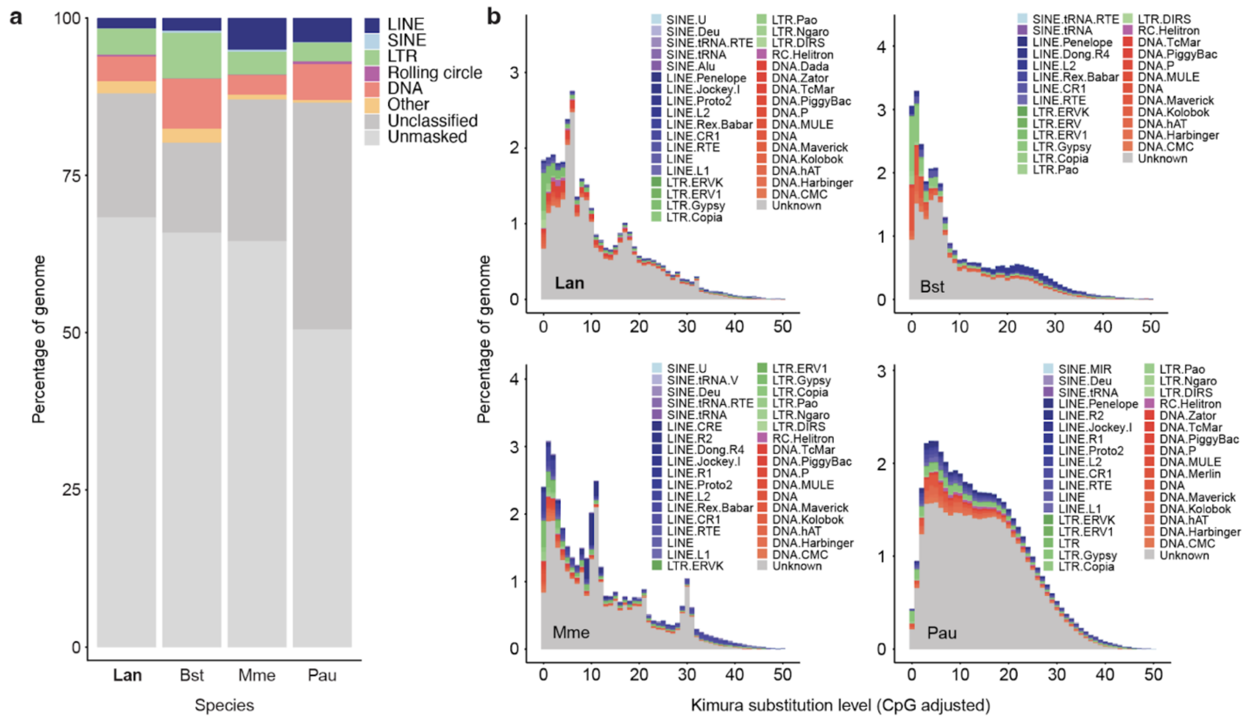

Supplementary Fig. 2 | Repeat content of Lophophorata genomes. a, Percent of genome assemblies composed of each repetitive element group for *Lingula anatina* (Lan), *Phoronis australis* (Pau), *Bugulina stolonifera* (Bst) and *Membranipora membranacea* (Mme) genomes. ‘Other’ includes Penelope elements, satellites, simple repeats and low complexity elements. *L. anatina* and the two bryozoans (*B. stolonifera* and *M. membranacea*) have highly similar repeat content composition. **b**, Output from RepeatLandscape. Plots show Kimura substitution level against percent of genome occupied and are coloured by transposable element taxonomy. Kimura substitution level is a proxy for transposable element age; the higher the substitution, the older the element insertion. There are recent repeat expansions in all Lophophorata genomes. In *L. anatina* and both bryozoans, a large proportion of the annotated elements with recent expansions are long terminal repeats (LTRs).

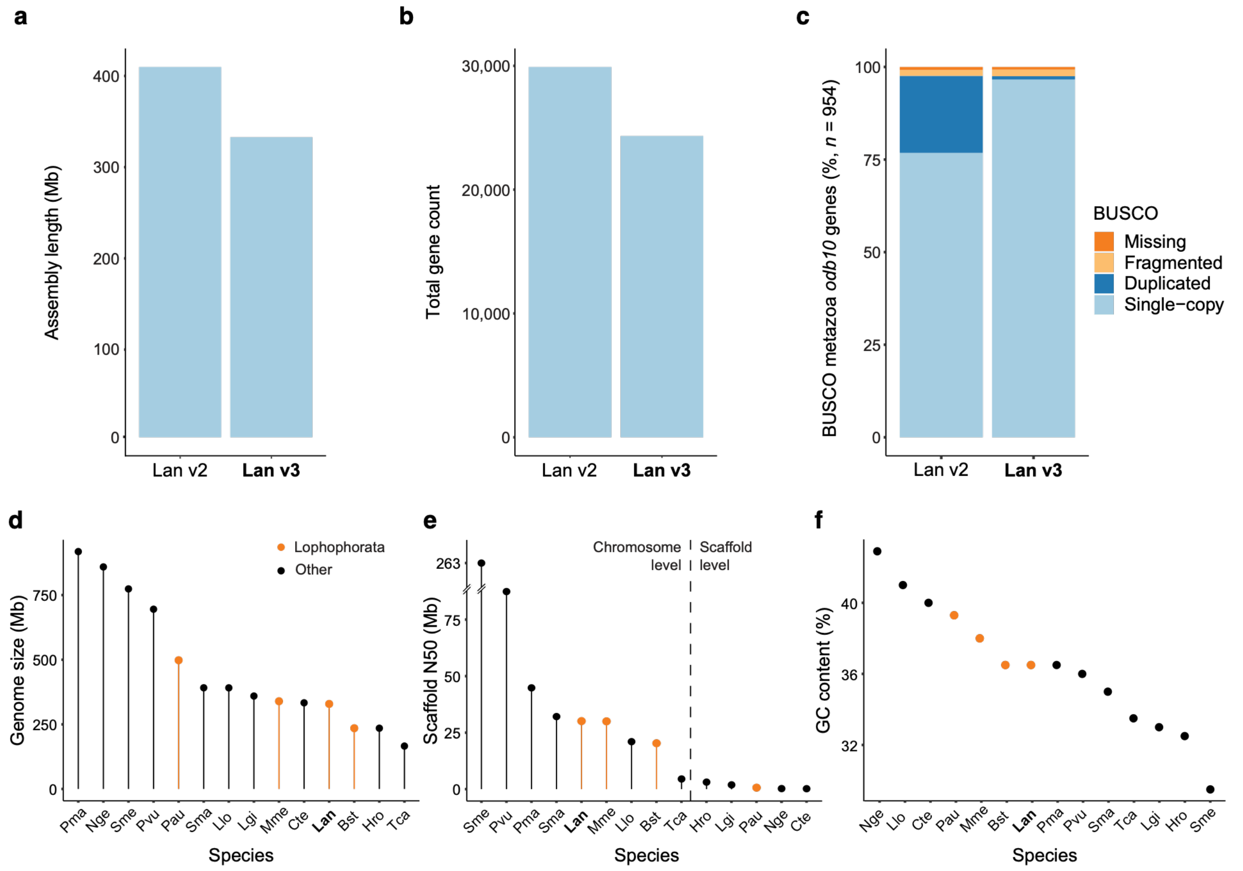

Supplementary Fig. 3 | Comparison between chromosome-level (this study) and draft assembly of the *L. anatina* genome. a, Total assembly lengths for the current (Lan v3) and previous (Lan v2) *L. anatina* assemblies (329 Mb versus 406 Mb). **b**, Total gene counts for current and previous *L. anatina* assemblies (24,330 versus 29,907). **c**, BUSCO Metazoa *odb10* for the current and previous *L. anatina* assemblies (0.9% duplication versus 20.8% duplication). Lower duplication, total gene count and shorter assembly lengths of the current assembly versus previous suggest false heterotype duplications were present in the previous assembly but are corrected in the current one. **d**, Genome size of selected spiralian assemblies and the outgroup *Tribolium castaneum*. Species within the clade Lophophorata are highlighted in orange. **e**, Scaffold N50 of selected spiralian assemblies. Species within the clade Lophophorata are highlighted in orange. **f**, GC content of selected spiralian assemblies. Species within the clade Lophophorata are highlighted in orange. Abbreviations: Bst, *Bugulina stolonifera*; Cte, *Capitella teleta*; Hro, *Helobdella robusta*; Lan, *Lingula anatina*; Lgi, *Lottia gigantea*; Llo, *Lineus longissimus*; Mme, *Membranipora membranacea*; Nge, *Notospermus geniculatus*; Pau, *Phoronis australis*; Pma, *Pecten maximus*; Pvu, *Patella vulgata*; Sma, *Schistosoma mansoni*; Sme, *Schmidtea mediterranea*; Tca, *Tribolium castaneum*.

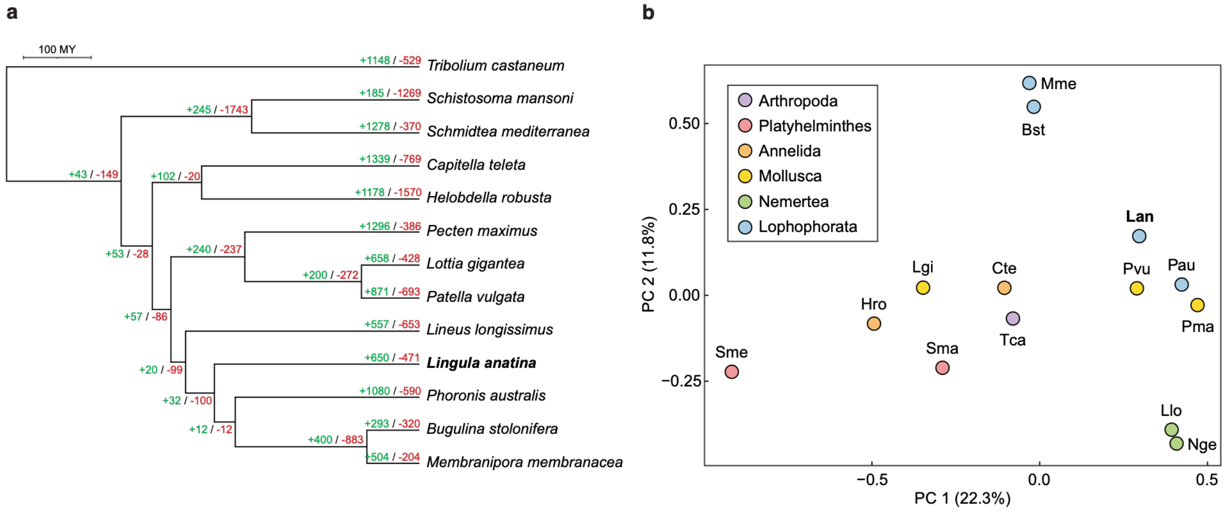

Supplementary Fig. 4 | Gene family evolution in spiralians. a, Output of CAFE analysis. Figures in green (+) and red (–)are estimated gene gains and losses, respectively. The brachiopod *L. anatina* has a relatively conservatively evolving gene content compared to other Lophophorata members: phoronids (*Phoronis australis*) have a large number of gene gains, while bryozoans (*Bugulina stolonifera* and *Membranipora membranacea*) have a high rate of gene loss. **b**, Principal component analysis (PCA) of spiralian gene content. OrthoFinder was used to create an orthogroup count matrix. The gene content of *L. anatina* (Lan) is most similar to that of phoronids (Pau) and molluscs (Pvu and Pma). The platyhelminths *Schmidtea mediterranea* and *Schistosoma mansoni* (Sme and Sma), the annelid *Helobdella robusta* (Hro) and the bryozoans *B. stolonifera* and *M. membranacea* all have extensive gene loss. The separate grouping of the two bryozoans away from the platyhelminths and annelid suggest different gene sets have been lost in these two groups. Abbreviations as in Supplementary Fig. 3.

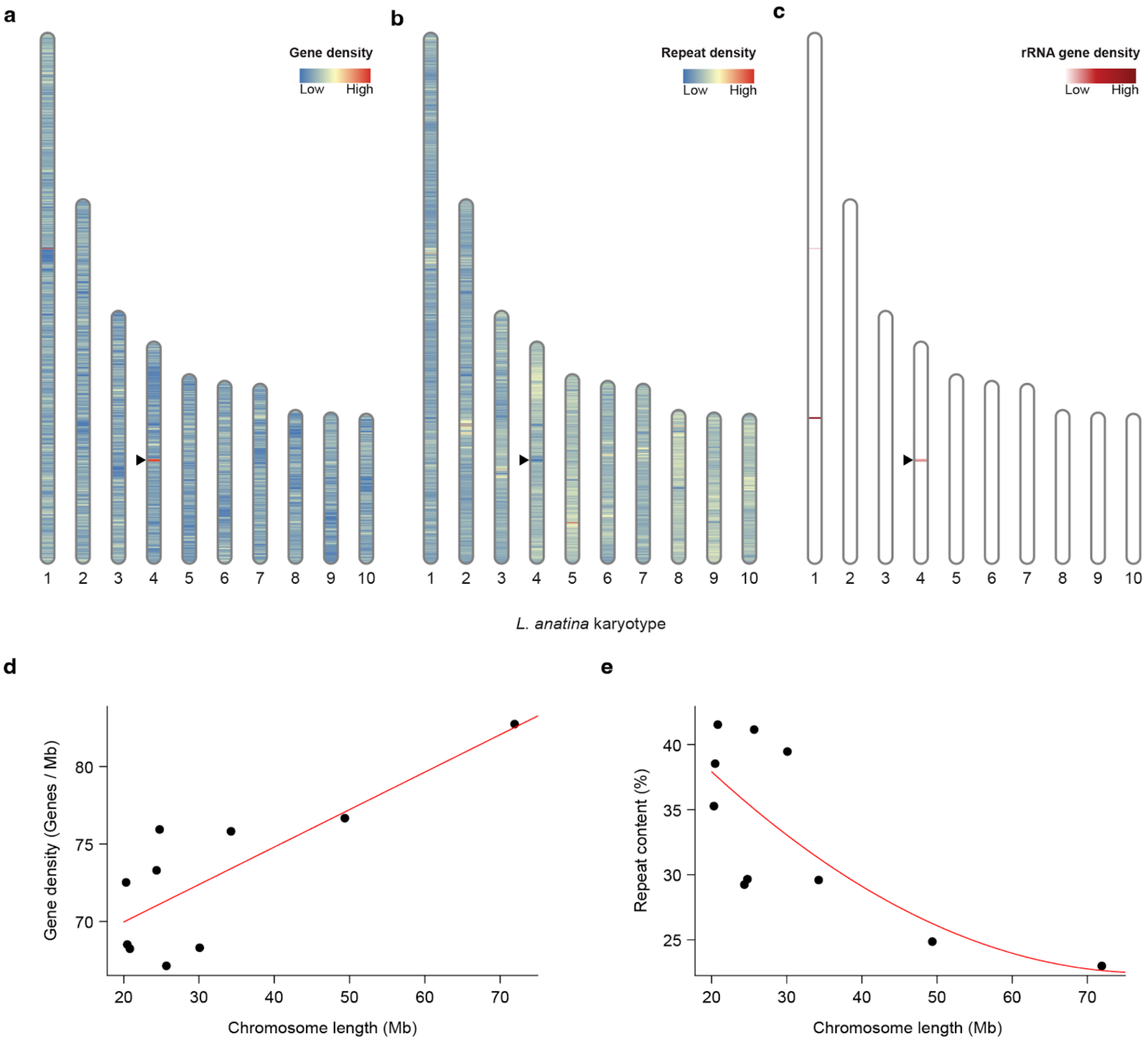

Supplementary Fig. 5 | Feature density in the *L. anatina* genome. Vertical bars represent chromosomes. Colours reflect feature density. Density plots were created with RIdeogram using windows of 100 kb. Black arrowheads mark a region on chromosome 4 with exceptionally high gene density and low repeat density; this region is a cluster of ribosomal RNA (rRNA) genes. **a**, Gene density in the *L. anatina* genome. **b**, Repeat density in the *L. anatina* genome. **c**, rRNA gene density in the *L. anatina* genome. The genomic region on chromosome 4 with high gene density and low repeat density is an rRNA gene cluster. **d**, Gene density plotted against chromosome length in *L. anatina*. Longer chromosomes have higher gene density (R-squared = 0.593, F-statistic = 14.120, *p*-value = 0.006). **e**, Repeat content plotted against chromosome length in *L. anatina*. Longer chromosomes have a non-significant lower percentage of repeats (R-squared = 0.441, F-statistic = 4.556, *p*-value = 0.054).

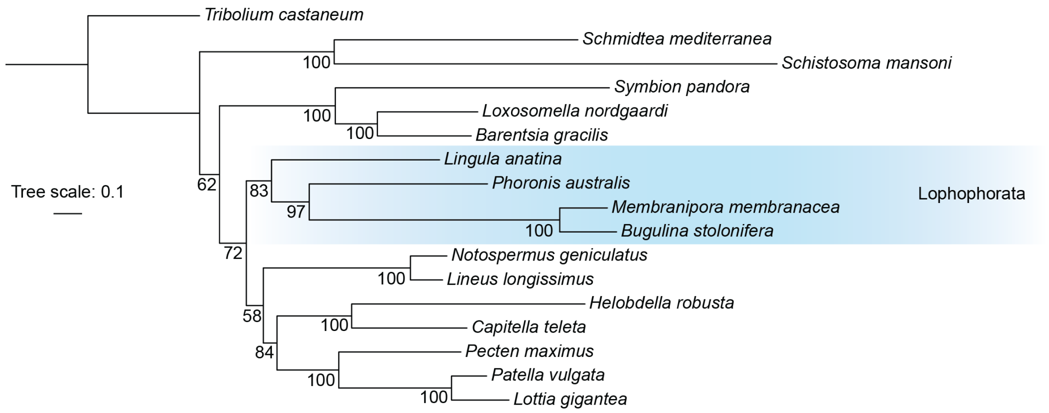

Supplementary Fig. 6 | Maximum likelihood tree containing Entoprocta and Cycliophora. Scale bar = amino acid substitutions per site. Tree constructed from 134 single-copy orthologues in IQ-TREE using 1,000 bootstrap replicates.

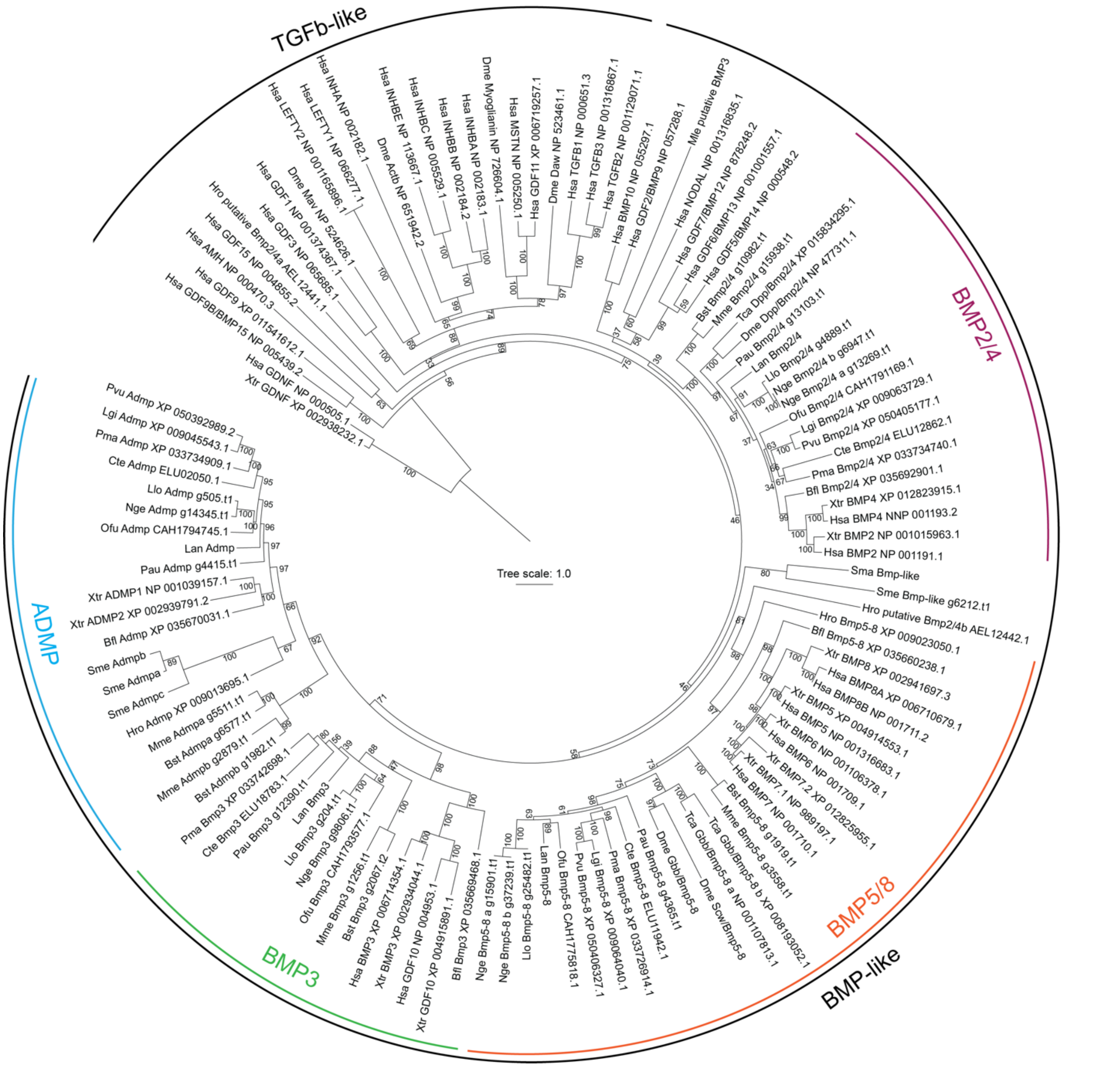

Supplementary Fig. 7 | Maximum likelihood tree of BMP ligand protein sequences identified in this work built using the Q.pfam+R7 model in IQ-TREE. Scale bar = amino acid substitutions per site. Numerical values = bootstrap support (1000 replicates). This work characterised Bmp2/4, Bmp3, Bmp5-8 and Admp ligands in *L. anatina* and other spiralian species. All human (*n* = 33) and *Drosophila* (*n* = 7) TGF-beta ligands are included as reference, including those from both TGFB-like and BMP-like families. Abbreviations: Bfl, *Branchiostoma floridae* (Chordata); Bst, *Bugulina stolonifera* (Bryozoa); Cte, *Capitella teleta* (Annelida) Dme, *Drosophila melanogaster* (Arthropoda); Hro, *Helobdella robusta* (Annelida) Hsa, *Homo sapiens* (Chordata); Lan, *Lingula anatina* (Brachiopoda); Lgi, *Lottia gigantea* (Mollusca); Llo, *Lineus longissimus* (Nemertea); Mme, *Membranipora membranacea* (Bryozoa); Nge, *Notospermus geniculatus* (Nemertea); Ofu, *Owenia fusiformis* (Annelida); Pau, *Phoronis australis* (Phoronida); Pma, *Pecten maximus* (Mollusca); Pvu, *Patella vulgata* (Mollusca); Sma, *Schistosoma mansoni*

(Platyhelminthes); Sme, *Schmidtea mediterranea* (Platyhelminthes); Tca, *Tribolium castaneum* (Arthropoda); Xtr, *Xenopus tropicalis* (Chordata).

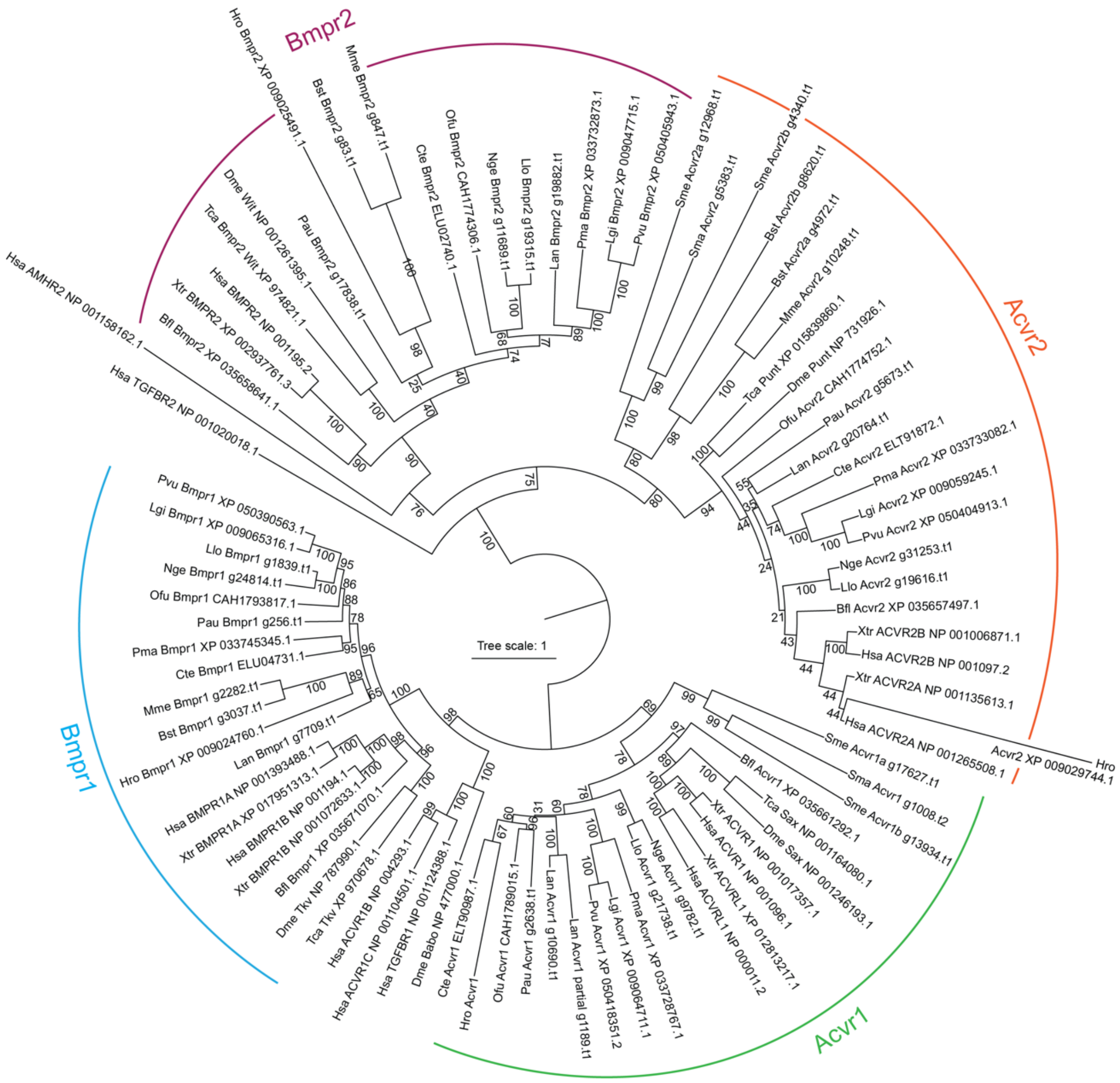

Supplementary Fig. 8 | Maximum likelihood tree of BMP receptor serine/threonine kinase protein sequences identified in this work built using the Q.pfam+R7 model in IQ-TREE. Scale bar = amino acid substitutions per site. Numerical values = bootstrap support (1000 replicates). This work characterised Acvr1, Acvr2, Bmpr1 and Bmpr2 in *L. anatina* and other spiralian species. All human (n = 12) and *Drosophila* (n = 5) receptor serine/threonine kinases are included as a reference, including those predominantly involved in TGF-beta signalling. Abbreviations: Bfl, *Branchiostoma floridae* (Chordata); Bst, *Bugulina stolonifera* (Bryozoa); Cte, *Capitella teleta* (Annelida) Dme, *Drosophila melanogaster* (Arthropoda); Hro, *Helobdella robusta* (Annelida) Hsa, *Homo sapiens* (Chordata); Lan, *Lingula anatina* (Brachiopoda); Lgi, *Lottia gigantea* (Mollusca); Llo, *Lineus longissimus* (Nemertea); Mme, *Membranipora membranacea* (Bryozoa); Nge, *Notospermus geniculatus* (Nemertea); Ofu, *Owenia fusiformis* (Annelida); Pau, *Phoronis australis* (Phoronida); Pma, *Pecten maximus* (Mollusca); Pvu, *Patella vulgata* (Mollusca); Sma, *Schistosoma mansoni* (Platyhelminthes); Sme, *Schmidtea mediterranea* (Platyhelminthes); Tca, *Tribolium castaneum* (Arthropoda); Xtr, *Xenopus tropicalis* (Chordata).

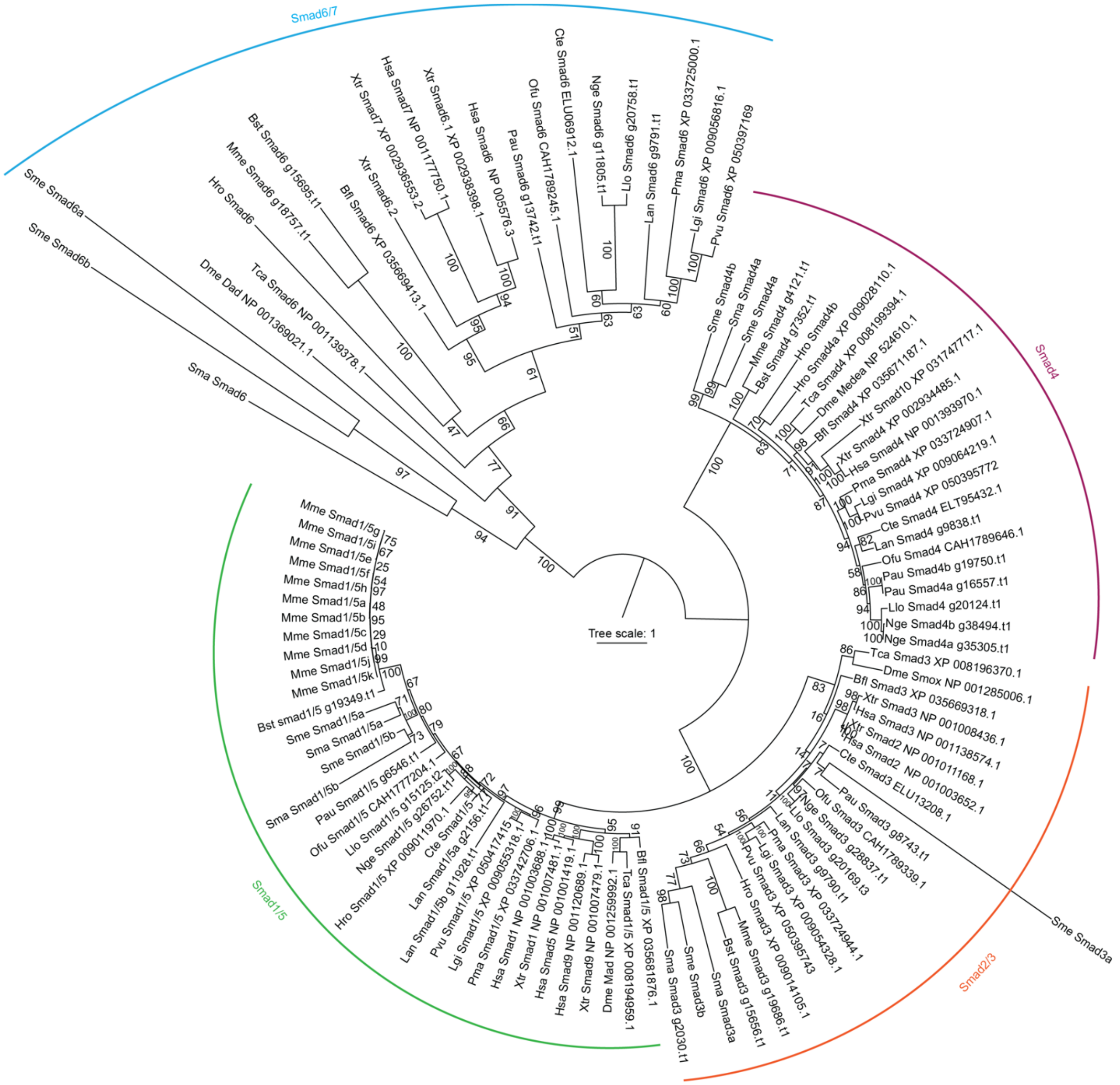

Supplementary Fig. 9 | Maximum likelihood tree of Smad protein sequences identified in this work built using the Q.insect+F+I+R6 model in IQ-TREE. Scale bar = amino acid substitutions per site. Numerical values = bootstrap support (1000 replicates). This work characterised Smad1/5, Smad2/3, Smad4 and Smad6/7 in *L. anatina* and other spiralian species. Abbreviations: Bfl, *Branchiostoma floridae* (Chordata); Bst, *Bugulina stolonifera* (Bryozoa); Cte, *Capitella teleta* (Annelida) Dme, *Drosophila melanogaster* (Arthropoda); Hro, *Helobdella robusta* (Annelida) Hsa, *Homo sapiens* (Chordata); Lan, *Lingula anatina* (Brachiopoda); Lgi, *Lottia gigantea* (Mollusca); Llo, *Lineus longissimus* (Nemertea); Mme, *Membranipora membranacea* (Bryozoa); Nge, *Notospermus geniculatus* (Nemertea); Ofu, *Owenia fusiformis* (Annelida); Pau, *Phoronis australis* (Phoronida); Pma, *Pecten maximus* (Mollusca); Pvu, *Patella vulgata* (Mollusca); Sma, *Schistosoma mansoni* (Platyhelminthes); Sme, *Schmidtea mediterranea* (Platyhelminthes); Tca, *Tribolium castaneum* (Arthropoda); Xtr, *Xenopus tropicalis* (Chordata).

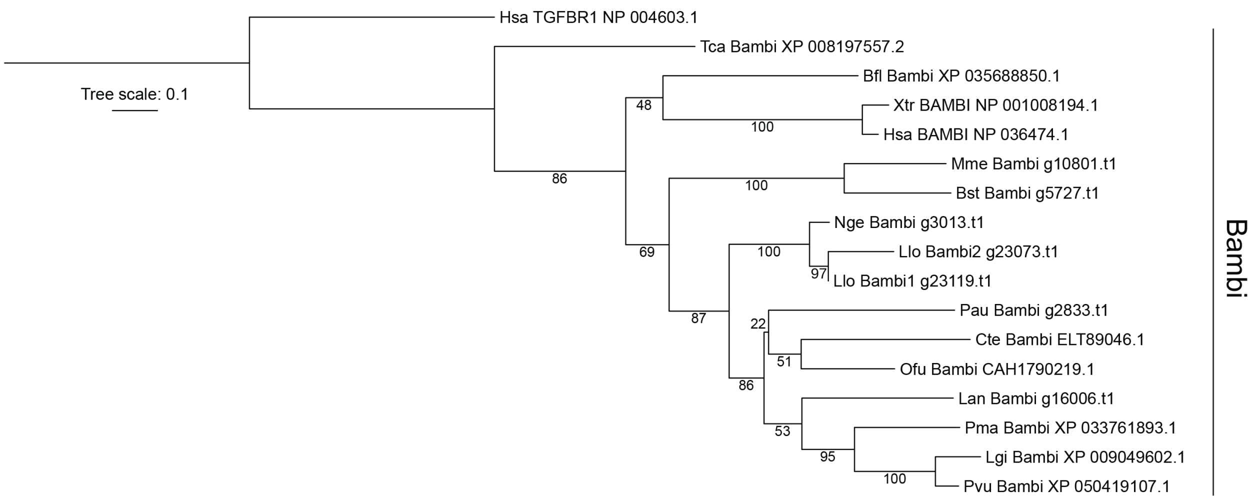

Supplementary Fig. 10 | Maximum likelihood tree of Bambi protein sequences identified in this work built using the VT+R3 model in IQ-TREE. Scale bar = amino acid substitutions per site. Numerical values = bootstrap support (1000 replicates). Abbreviations: Bfl, *Branchiostoma floridae* (Chordata); Bst, *Bugulina stolonifera* (Bryozoa); Cte, *Capitella teleta* (Annelida) Dme, *Drosophila melanogaster* (Arthropoda); Hro, *Helobdella robusta* (Annelida) Hsa, *Homo sapiens* (Chordata); Lan, *Lingula anatina* (Brachiopoda); Lgi, *Lottia gigantea* (Mollusca); Llo, *Lineus longissimus* (Nemertea); Mme, *Membranipora membranacea* (Bryozoa); Nge, *Notospermus geniculatus* (Nemertea); Ofu, *Owenia fusiformis* (Annelida); Pau, *Phoronis australis* (Phoronida); Pma, *Pecten maximus* (Mollusca); Pvu, *Patella vulgata* (Mollusca); Sma, *Schistosoma mansoni* (Platyhelminthes); Sme, *Schmidtea mediterranea* (Platyhelminthes); Tca, *Tribolium castaneum* (Arthropoda); Xtr, *Xenopus tropicalis* (Chordata).

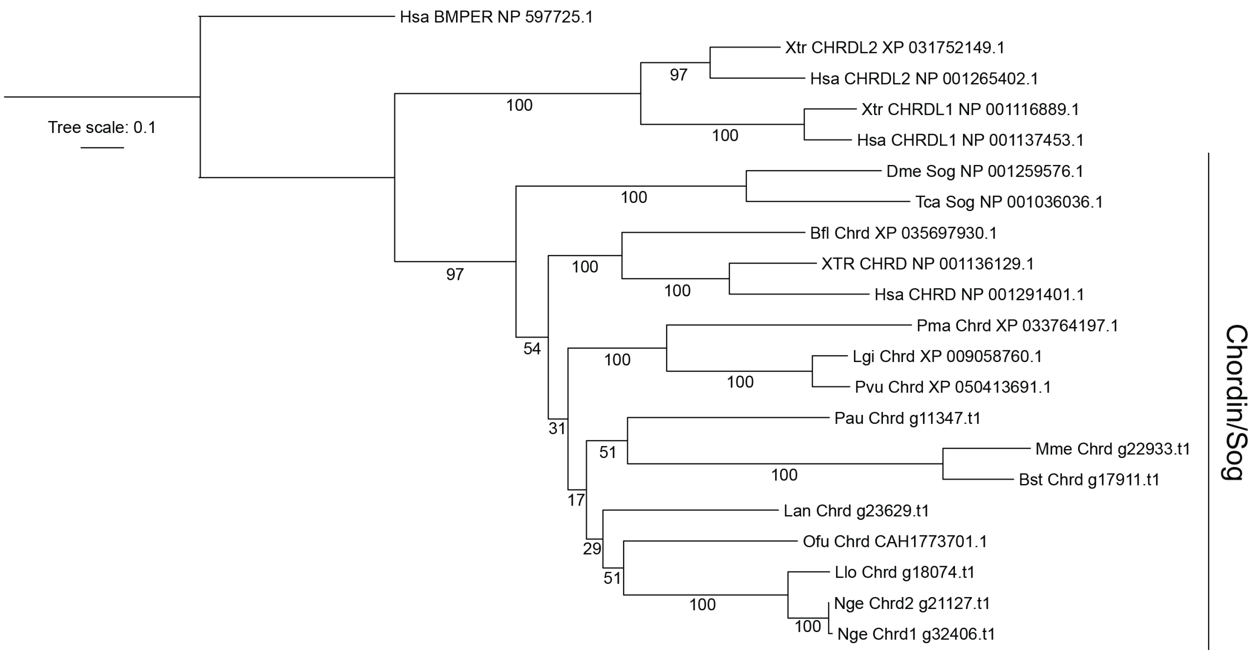

Supplementary Fig. 11 | Maximum likelihood tree of Chordin/Sog protein sequences identified in this work built using the WAG+I+G4 model in IQ-TREE. Scale bar = amino acid substitutions per site. Numerical values = bootstrap support (1000 replicates). Abbreviations: Bfl, *Branchiostoma floridae* (Chordata); Bst, *Bugulina stolonifera* (Bryozoa); Cte, *Capitella teleta* (Annelida) Dme, *Drosophila melanogaster* (Arthropoda); Hro, *Helobdella robusta* (Annelida) Hsa, *Homo sapiens* (Chordata); Lan, *Lingula anatina* (Brachiopoda); Lgi, *Lottia gigantea* (Mollusca); Llo, *Lineus longissimus* (Nemertea); Mme, *Membranipora membranacea* (Bryozoa); Nge, *Notospermus geniculatus* (Nemertea); Ofu, *Owenia fusiformis* (Annelida); Pau, *Phoronis australis* (Phoronida); Pma, *Pecten maximus* (Mollusca); Pvu, *Patella vulgata* (Mollusca); Sma, *Schistosoma mansoni* (Platyhelminthes); Sme, *Schmidtea mediterranea* (Platyhelminthes); Tca, *Tribolium castaneum* (Arthropoda); Xtr, *Xenopus tropicalis* (Chordata).

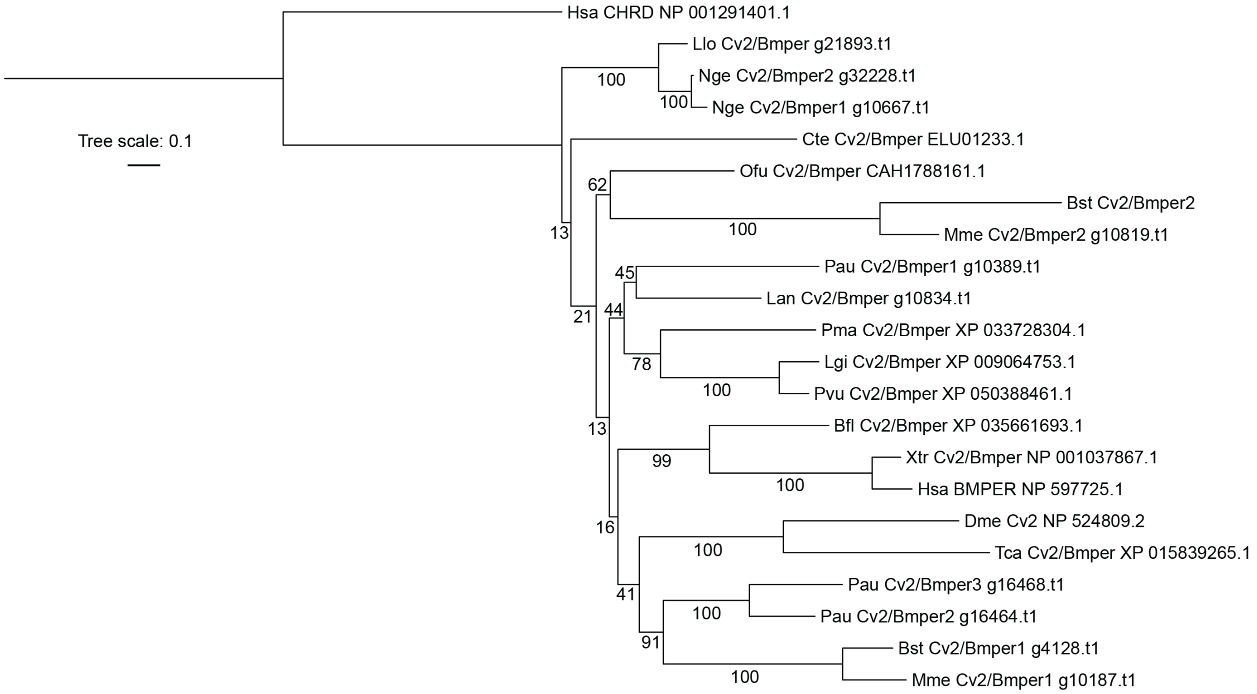

Supplementary Fig. 12 | Maximum likelihood tree of Cv2/Bmper protein sequences identified in this work built using the WAG+R4 model in IQ-TREE. Scale bar = amino acid substitutions per site. Numerical values = bootstrap support (1000 replicates). Abbreviations: Bfl, *Branchiostoma floridae* (Chordata); Bst, *Bugulina stolonifera* (Bryozoa); Cte, *Capitella teleta* (Annelida) Dme, *Drosophila melanogaster* (Arthropoda); Hro, *Helobdella robusta* (Annelida) Hsa, *Homo sapiens* (Chordata); Lan, *Lingula anatina* (Brachiopoda); Lgi, *Lottia gigantea* (Mollusca); Llo, *Lineus longissimus* (Nemertea); Mme, *Membranipora membranacea* (Bryozoa); Nge, *Notospermus geniculatus* (Nemertea); Ofu, *Owenia fusiformis* (Annelida); Pau, *Phoronis australis* (Phoronida); Pma, *Pecten maximus* (Mollusca); Pvu, *Patella vulgata* (Mollusca); Sma, *Schistosoma mansoni* (Platyhelminthes); Sme, *Schmidtea mediterranea* (Platyhelminthes); Tca, *Tribolium castaneum* (Arthropoda); Xtr, *Xenopus tropicalis* (Chordata).

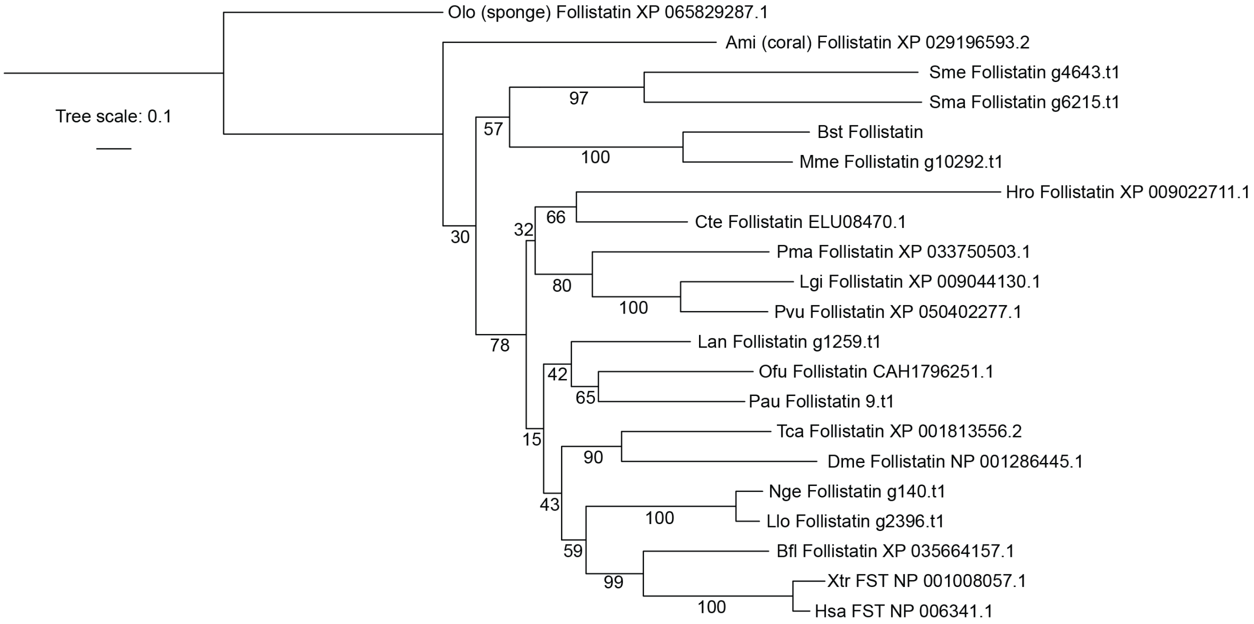

Supplementary Fig. 13 | Maximum likelihood tree of Follistatin protein sequences identified in this work built using the WAG+I+G4 model in IQ-TREE. Scale bar = amino acid substitutions per site. Numerical values = bootstrap support (1000 replicates). Abbreviations: Ami, *Acropora millepora* (Cnidaria); Bfl, *Branchiostoma floridae* (Chordata); Bst, *Bugulina stolonifera* (Bryozoa); Cte, *Capitella teleta* (Annelida) Dme, *Drosophila melanogaster* (Arthropoda); Hro, *Helobdella robusta* (Annelida) Hsa, *Homo sapiens* (Chordata); Lan, *Lingula anatina* (Brachiopoda); Lgi, *Lottia gigantea* (Mollusca); Llo, *Lineus longissimus* (Nemertea); Mme, *Membranipora membranacea* (Bryozoa); Nge, *Notospermus geniculatus* (Nemertea); Ofu, *Owenia fusiformis* (Annelida); Olo, *Oscarella lobularis* (Porifera); Pau, *Phoronis australis* (Phoronida); Pma, *Pecten maximus* (Mollusca); Pvu, *Patella vulgata* (Mollusca); Sma, *Schistosoma mansoni* (Platyhelminthes); Sme, *Schmidtea mediterranea* (Platyhelminthes); Tca, *Tribolium castaneum* (Arthropoda); Xtr, *Xenopus tropicalis* (Chordata).

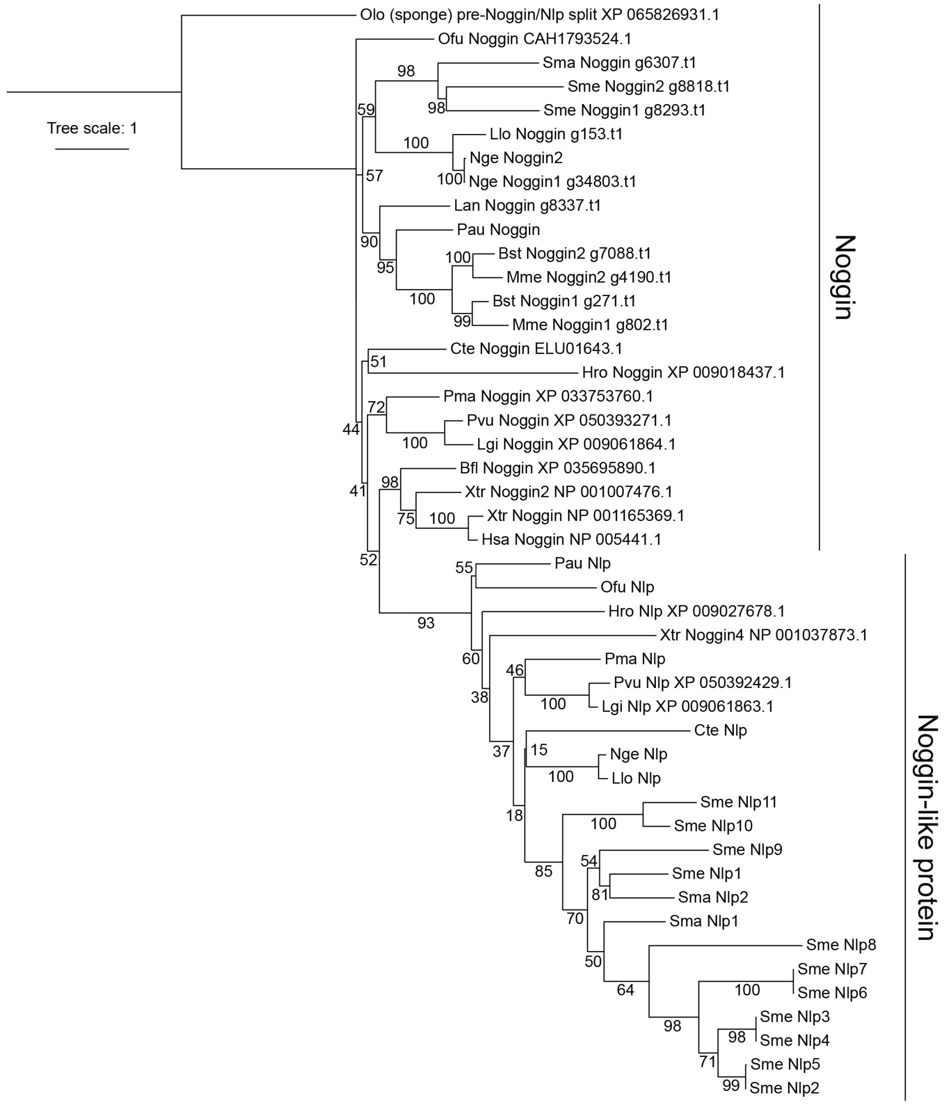

Supplementary Fig. 14 | Maximum likelihood tree of Noggin-like protein and Noggin protein sequences identified in this work built using the Q.pfam+R4 model in IQ-TREE. Scale bar = amino acid substitutions per site. Numerical values = bootstrap support (1000 replicates). Abbreviations: Bfl, *Branchiostoma floridae* (Chordata); Bst, *Bugulina stolonifera* (Bryozoa); Cte, *Capitella teleta* (Annelida) Dme, *Drosophila melanogaster* (Arthropoda); Hro, *Helobdella robusta* (Annelida) Hsa, *Homo sapiens* (Chordata); Lan, *Lingula anatina* (Brachiopoda); Lgi, *Lottia gigantea* (Mollusca); Llo, *Lineus longissimus* (Nemertea); Mme, *Membranipora membranacea* (Bryozoa); Nge, *Notospermus geniculatus* (Nemertea); Ofu, *Owenia fusiformis* (Annelida); Olo, *Oscarella lobularis* (Porifera); Pau, *Phoronis australis* (Phoronida); Pma, *Pecten maximus* (Mollusca); Pvu, *Patella vulgata* (Mollusca); Sma, *Schistosoma mansoni* (Platyhelminthes); Sme, *Schmidtea mediterranea* (Platyhelminthes); Tca, *Tribolium castaneum* (Arthropoda); Xtr, *Xenopus tropicalis* (Chordata).

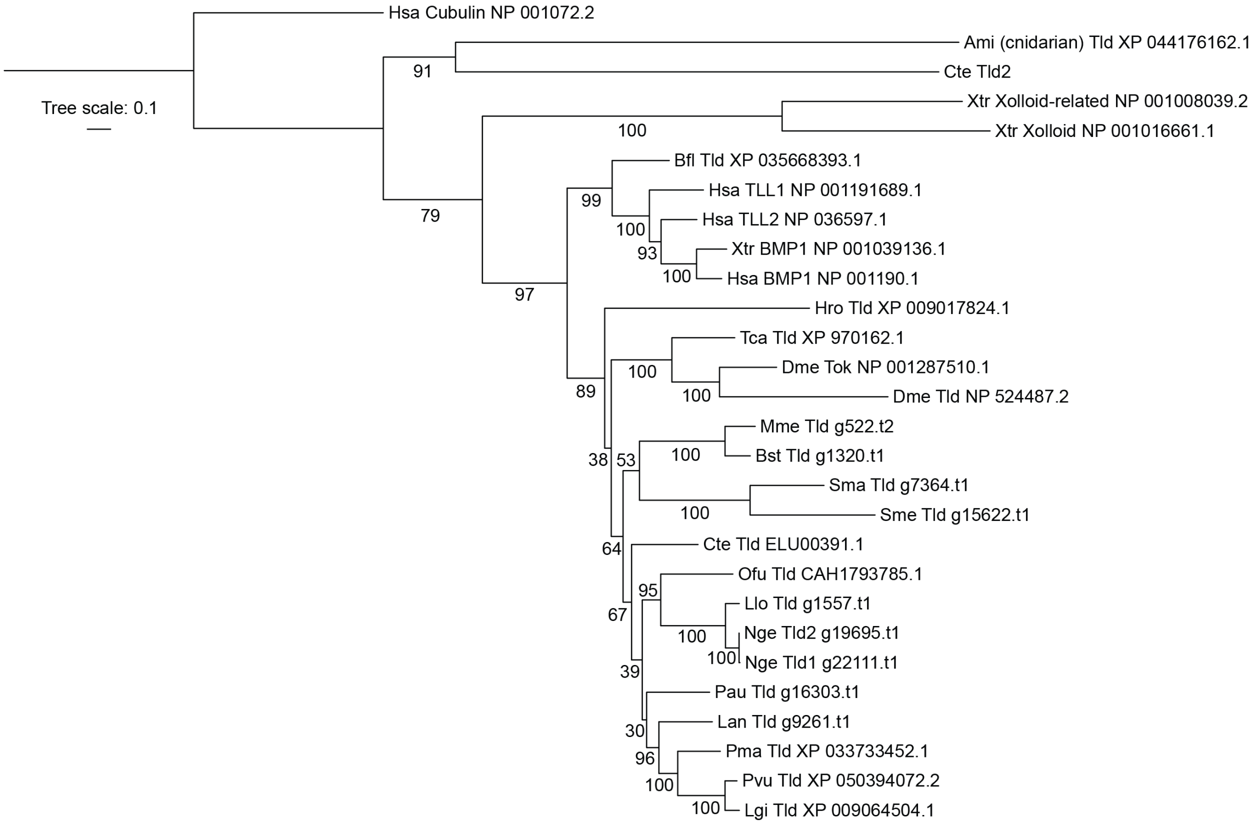

Supplementary Fig. 15 | Maximum likelihood tree of Bmp1/Tolloid protein sequences identified in this work built using the LG+G4 model in IQ-TREE. Scale bar = amino acid substitutions per site. Numerical values = bootstrap support (1000 replicates). Abbreviations: Ami, *Acropora millepora* (Cnidaria); Bfl, *Branchiostoma floridae* (Chordata); Bst, *Bugulina stolonifera* (Bryozoa); Cte, *Capitella teleta* (Annelida) Dme, *Drosophila melanogaster* (Arthropoda); Hro, *Helobdella robusta* (Annelida) Hsa, *Homo sapiens* (Chordata); Lan, *Lingula anatina* (Brachiopoda); Lgi, *Lottia gigantea* (Mollusca); Llo, *Lineus longissimus* (Nemertea); Mme, *Membranipora membranacea* (Bryozoa); Nge, *Notospermus geniculatus* (Nemertea); Ofu, *Owenia fusiformis* (Annelida); Pau, *Phoronis australis* (Phoronida); Pma, *Pecten maximus* (Mollusca); Pvu, *Patella vulgata* (Mollusca); Sma, *Schistosoma mansoni* (Platyhelminthes); Sme, *Schmidtea mediterranea* (Platyhelminthes); Tca, *Tribolium castaneum* (Arthropoda); Xtr, *Xenopus tropicalis* (Chordata).

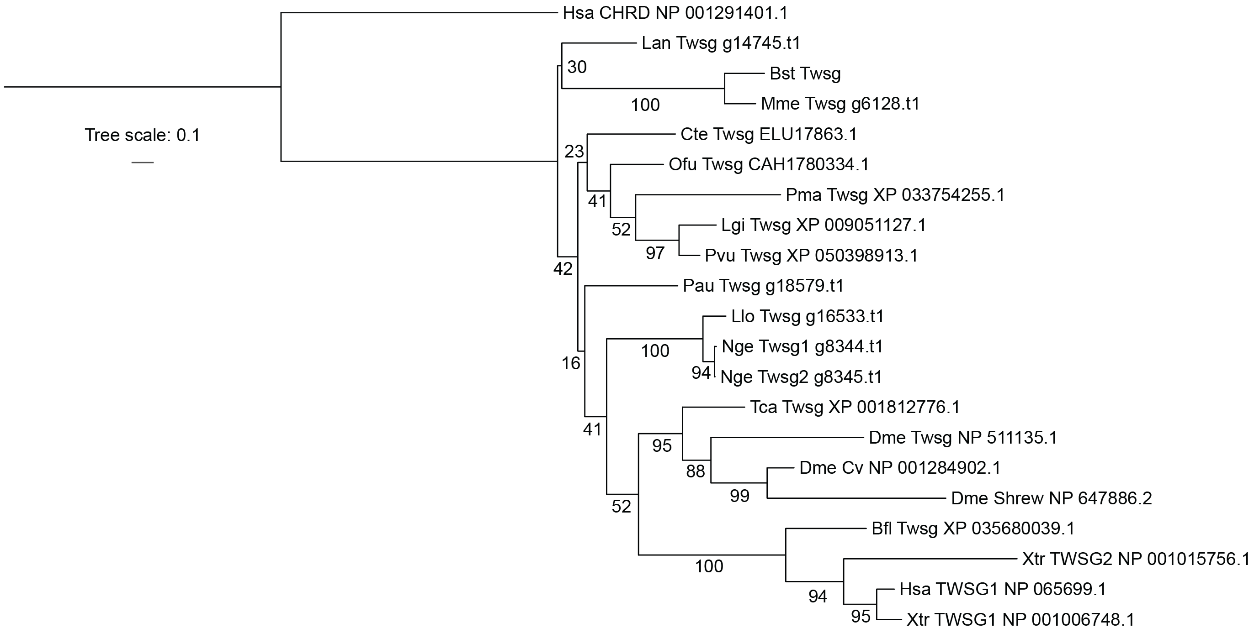

Supplementary Fig. 16 | Maximum likelihood tree of Twisted gastrulation protein sequences identified in this work built using the WAG+G4 model in IQ-TREE. Scale bar = amino acid substitutions per site. Numerical values = bootstrap support (1000 replicates). Abbreviations: Bfl, *Branchiostoma floridae* (Chordata); Bst, *Bugulina stolonifera* (Bryozoa); Cte, *Capitella teleta* (Annelida) Dme, *Drosophila melanogaster* (Arthropoda); Hro, *Helobdella robusta* (Annelida) Hsa, *Homo sapiens* (Chordata); Lan, *Lingula anatina* (Brachiopoda); Lgi, *Lottia gigantea* (Mollusca); Llo, *Lineus longissimus* (Nemertea); Mme, *Membranipora membranacea* (Bryozoa); Nge, *Notospermus geniculatus* (Nemertea); Ofu, *Owenia fusiformis* (Annelida); Pau, *Phoronis australis* (Phoronida); Pma, *Pecten maximus* (Mollusca); Pvu, *Patella vulgata* (Mollusca); Sma, *Schistosoma mansoni* (Platyhelminthes); Sme, *Schmidtea mediterranea* (Platyhelminthes); Tca, *Tribolium castaneum* (Arthropoda); Xtr, *Xenopus tropicalis* (Chordata).

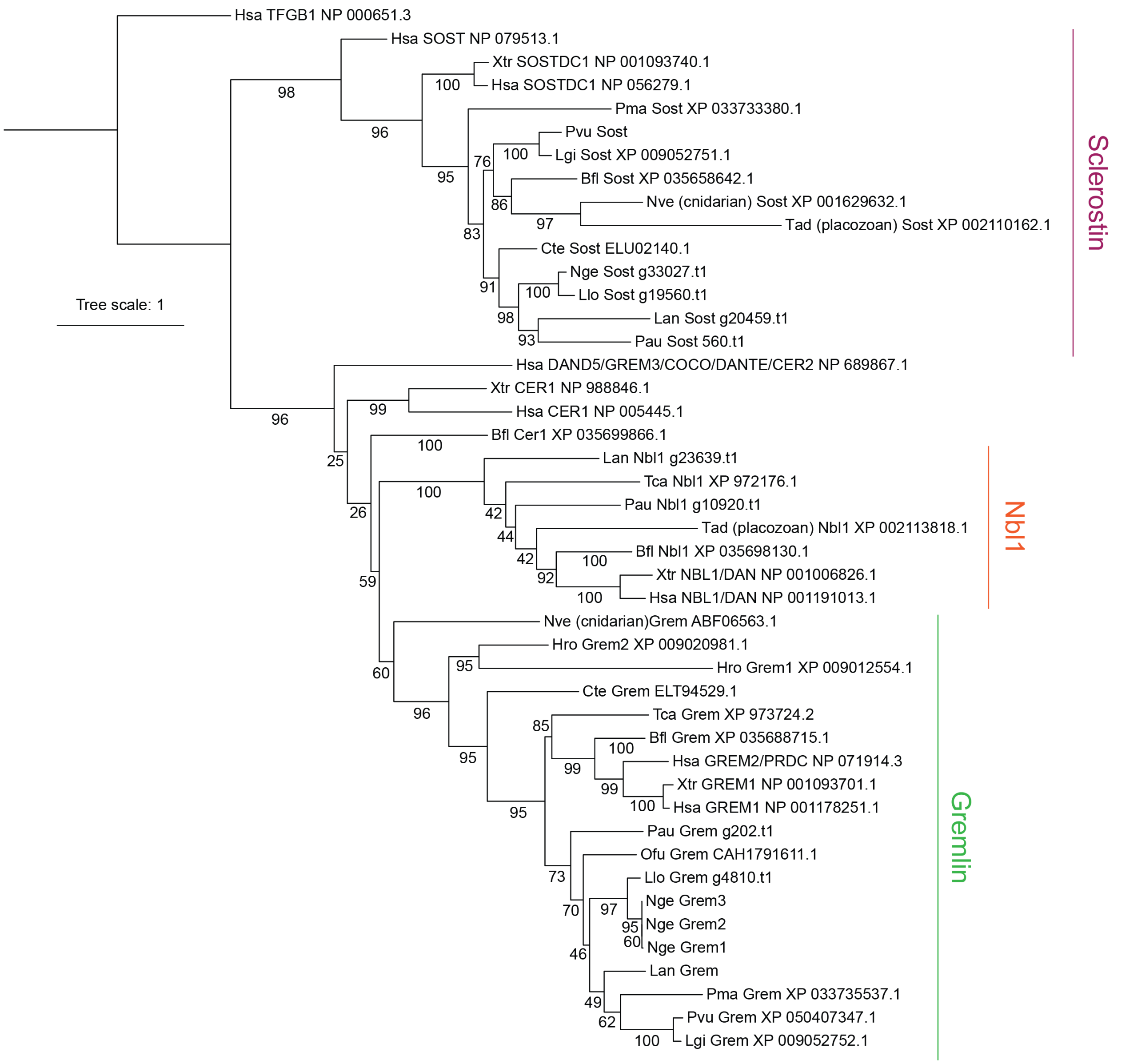

Supplementary Fig. 17 | Maximum likelihood tree of Dan family protein sequences identified in this work built using the VT+R5 model in IQ-TREE. This work characterised Gremlin, Nbl1, and Sclerostin in *L. anatina* and other spiralian species. Scale bar = amino acid substitutions per site. Numerical values = bootstrap support (1000 replicates). Abbreviations: Bfl, *Branchiostoma floridae* (Chordata); Cte, *Capitella teleta* (Annelida) Dme, *Drosophila melanogaster* (Arthropoda); Hro, *Helobdella robusta* (Annelida) Hsa, *Homo sapiens* (Chordata); Lan, *Lingula anatina* (Brachiopoda); Lgi, *Lottia gigantea* (Mollusca); Llo, *Lineus longissimus* (Nemertea); Nge, *Notospermus geniculatus* (Nemertea); Nve, *Nematostella vectensis* (Cnidaria); Ofu, *Owenia fusiformis* (Annelida); Pau, *Phoronis australis* (Phoronida); Pma, *Pecten maximus* (Mollusca); Pvu, *Patella vulgata* (Mollusca); Sma, *Schistosoma mansoni* (Platyhelminthes); Sme, *Schmidtea mediterranea* (Platyhelminthes); Tad, *Trichoplax adhaerens* (Placozoa); Tca, *Tribolium castaneum* (Arthropoda); Xtr, *Xenopus tropicalis* (Chordata).

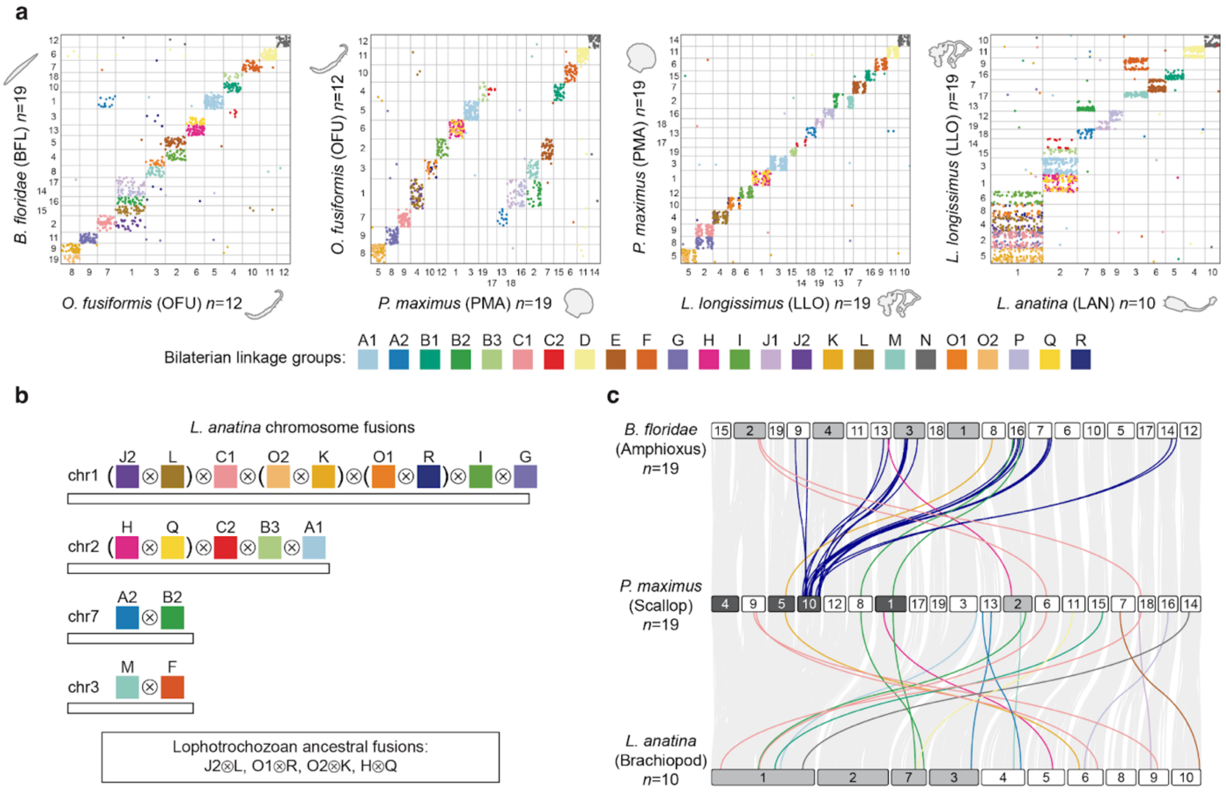

Supplementary Fig. 18 | Chromosome-scale gene linkage in spiralians. a, Oxford dot plots showing chromosome-scale gene linkage between the chordate *B. floridae*, annelid *O. fusiformis*, mollusc *P. maximus*, nemertean *L. longissimus* and brachiopod *L. anatina*. Each axis represents the entire length of the genome of one species. Grey bars separate chromosomes. Each point represents a pair of orthologues, placed by their ordinal position in each genome and coloured by the bilaterian ALG to which they belong. *L. anatina* chromosome 1 corresponds to five *L. longissimus* chromosomes (chr 2, 4, 5, 6, 8). *L. anatina* chromosome 2 corresponds to four *L. longissimus* chromosomes (chr 1, 3, 14, 15). *L. anatina* chromosome 7 corresponds to two *L. longissimus* chromosomes (chr 13, 18). *L. anatina* chromosome 3 corresponds to two *L. longissimus* chromosomes (chr 9, 17). **b,** Summary of chromosome fusion events inferred from the *L. anatina* genome. The symbol ⊗ represents a fusion-with-mixing event. Fusions that are present not only in brachiopods but also in other lophotrochozoans (annelids, molluscs and nemerteans) are highlighted with brackets. *L. anatina* chromosome 1 hosts genes from nine ALGs (J2, L, C1, O2, K, O1, R, I, G). *L. anatina* chromosome 2 hosts genes from five ALGs (H, Q, C2, B3, A1). *L. anatina* chromosome 7 hosts genes from two ALGs (A2, B2). *L. anatina* chromosome 3 hosts genes from two ALGs (M, F). **c,** 'Genes of variable synteny' in the genomes of *B. floridae* (Chordata), *P. maximus* (Mollusca), and *L. anatina* (Brachiopoda). To create informative macrosynteny plots, cases where five or fewer genes are translocated to a chromosome composed of a different ALG are removed from the figures. This plot shows in such genes (i.e. the genes removed from main text Fig. 1b) in colour, while pale grey lines

represent genes with a conserved ALG, showing relationships between chromosomes. While most chromosomes have few translocations, *P. maximus* chromosome 10 hosts many genes in ALG R that appear to have been translocated in the *B. floridae* genome. This is because, as previously reported, ALG R has been completely lost in *B. floridae*, so remaining genes are dispersed around the genome.

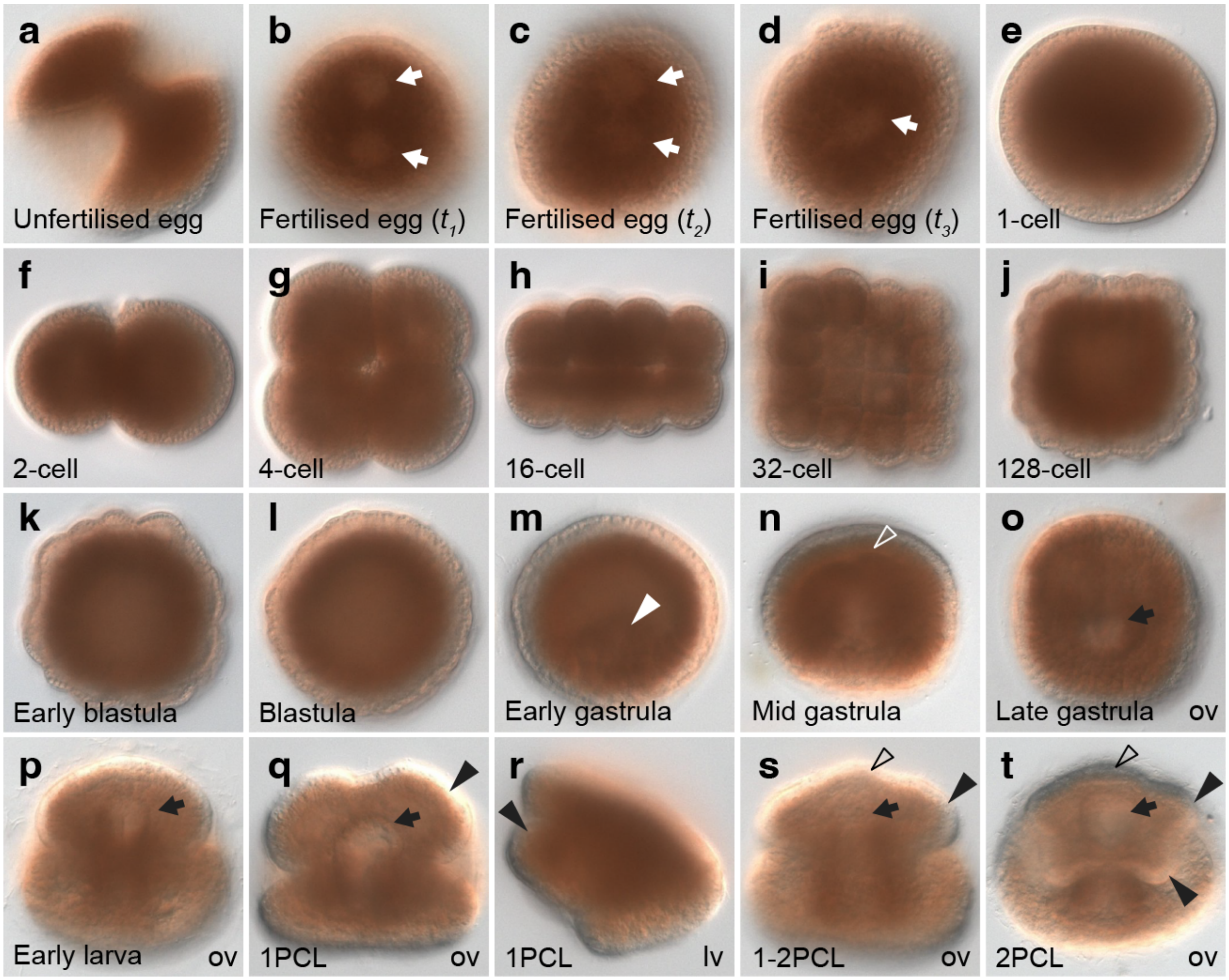

Supplementary Fig. 19 | Early embryonic development of the brachiopod *L. anatina*. a, Unfertilised eggs typically exhibit an irregular shape. **b**, Within 15 min post-insemination, these eggs transform dramatically into a more rounded form. **b**–**d**, This time sequence, from t_1 to t_3, illustrates the pronuclear fusion event post-fertilization. White arrows indicate the pronuclei. **e**–**t**, Early developmental stages from a single cell to the larval stage. **e**, 1-cell. **f**, 2-cell, **g**, 4-cell, **h**, 16-cell. **i**, 32-cell. **j**, 128-cell. **k**, Early blastula, distinguished by the presence of blastomeres. **l**, Blastula, where a blastoderm is formed. **m**, Early gastrula, starting with invagination (indicated by arrowhead). **n**, The mid gastrula, characterised by the archenteron contacting the ectoderm (shown by an empty arrowhead). **o**, Late gastrula, with the blastopore marked by an arrow. **p**, Early larva, identifiable by the absence of cirri. **q** and **r**, Larval stages with one pair of cirri (1PCL). **s**, Transitional stage from one to two pairs of cirri (1-2PCL), showing tentacle development. **t**, Larval stage with two pairs of cirri (2PCL). Arrows in these stages (**o**–**t**) denote the blastopore, which becomes the mouth area, while the arrowheads and blank arrowheads mark the cirri and tentacles, respectively. ov and lv represent oral and lateral views.

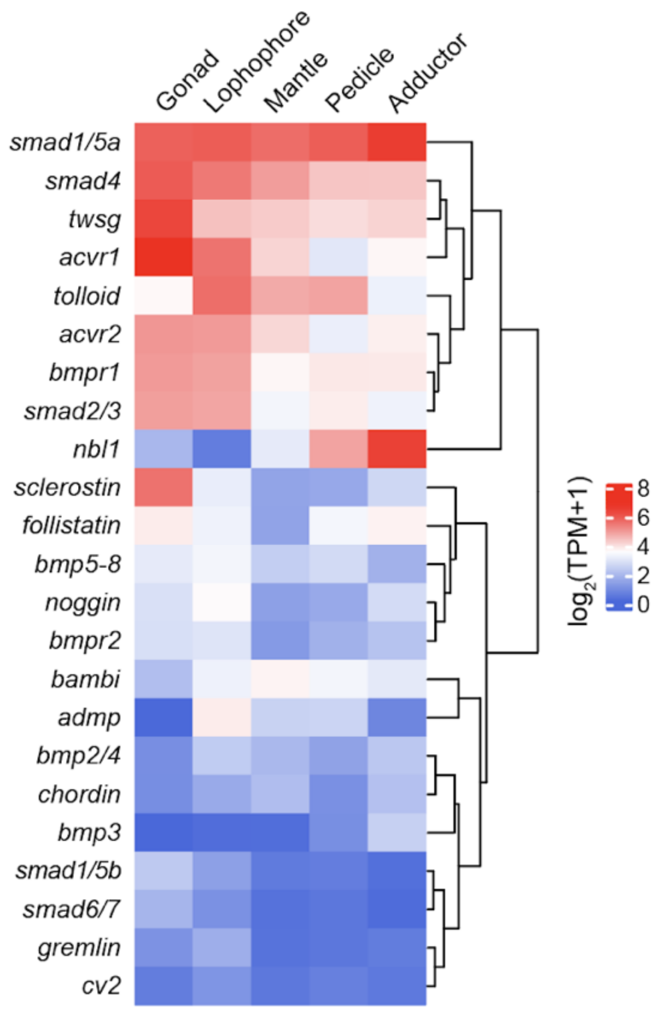

Supplementary Fig. 20 | Expression of BMP pathway genes in *L. anatina*. Expression profiles of BMP signalling ligands, mediators, and modulators during in five adult *L. anatina* tissues. TPM, transcripts per million.

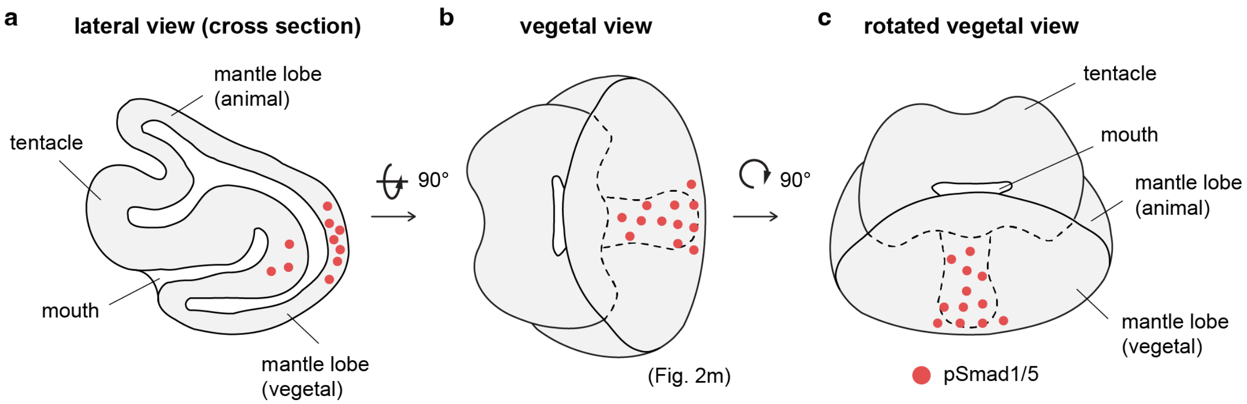

Supplementary Fig. 21 | Schematics of *L. anatina* embryos at the early larval stage.
a, Early larva in lateral view. **b**, One-pair-cirri larva in vegetal view. **c**, One-pair-cirri larva in rotated vegetal view. Phosphorylated Smad1/5 is marekd with red points.

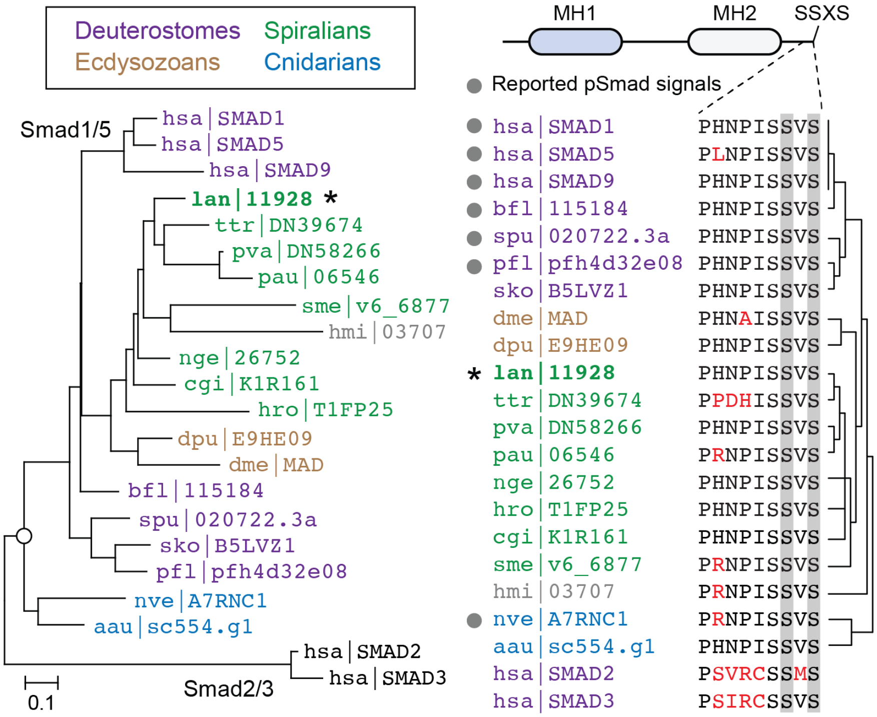

Supplementary Fig. 22 | Smad1/5 evolution and the phosphopeptide motif. Phylogeny of Smad1/5 genes inferred with 427 amino acid positions using the maximum likelihood method with the LG model and 1,000 bootstrap replications. Numbers at the nodes indicate bootstrap support values. Proteins are identified by their UniProt, gene model, or transcriptome IDs. *L. anatina* Smad1/5 is indicated with an asterisk. Alignment shows the C-terminus of Smad1/5 proteins. The PHNPISSVS peptide against which the antibody used in this work is active, which is found in human Smad1, is perfectly conserved in the Smad1/5 protein of *L. anatina*. Phosphorylated sites corresponding to Ser463/465 in human Smad1 are shaded in grey. Different amino acids compared to human Smad1 are labelled in red. Grey circles indicate reported pSmad1/5 signals using the antibody against the phosphopeptide PHNPISSVS. Species tree on the right.

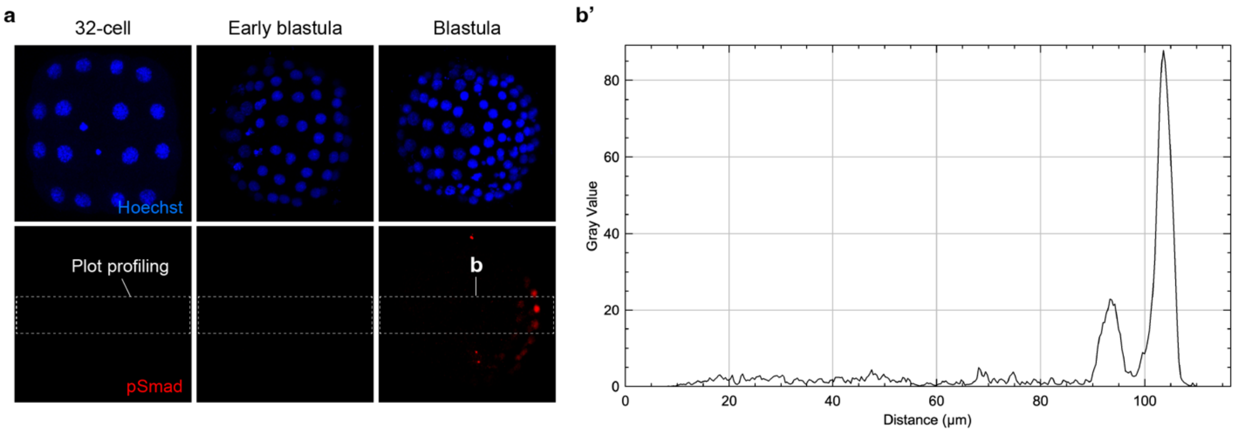

Supplementary Fig. 23 | Immunostaining with pSmad antibody and signal quantification. **a**, Immunostaining of *L. anatina* embryos using a pSmad1/5 antibody, with nuclear counterstaining by Hoechst 33342. Regions used for signal profiling are indicated with dashed boxes. **b**, Signal intensity profiling of pSmad staining in blastula-stage embryos, with quantification shown in b’ using ImageJ. pSmad signals were absent at both the 32-cell and early blastula stages.

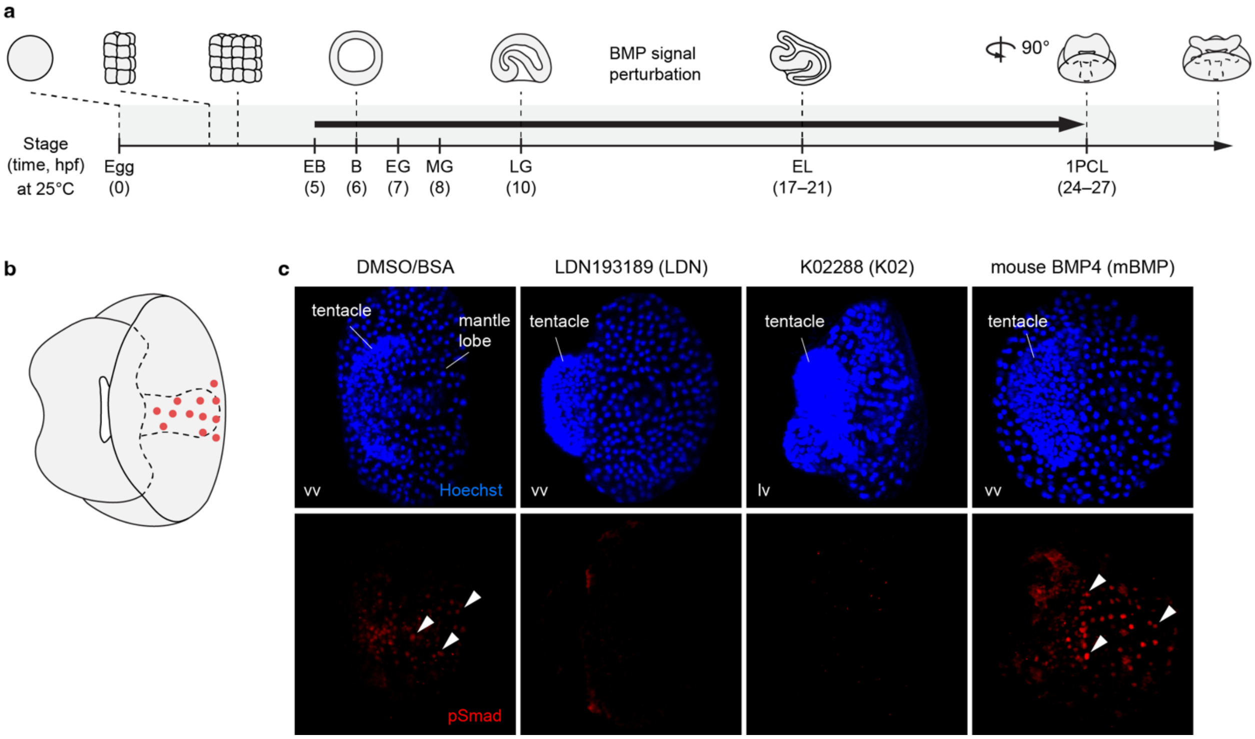

Supplementary Fig. 24 | BMP signalling readout in the larval stage detected by pSmad immunostaining. a, Schematic of the *L. anatina* developmental timeline (hours post-fertilisation, hpf) and experimental manipulation of BMP signalling from the early blastula (EB) to one-pair-cirri larval (1PCL) stage. B, blastula; EG, early gastrula; MG, mid-gastrula; LG, late gastrula; EL, early larva. **b**, Vegetal view of a control 1PCL larva, with the location of pSmad1/5 staining marked in red. **c**, Immunostaining of *L. anatina* embryos treated with small-molecule inhibitors or recombinant proteins, using a pSmad1/5 antibody and Hoechst 33342 nuclear counterstain.

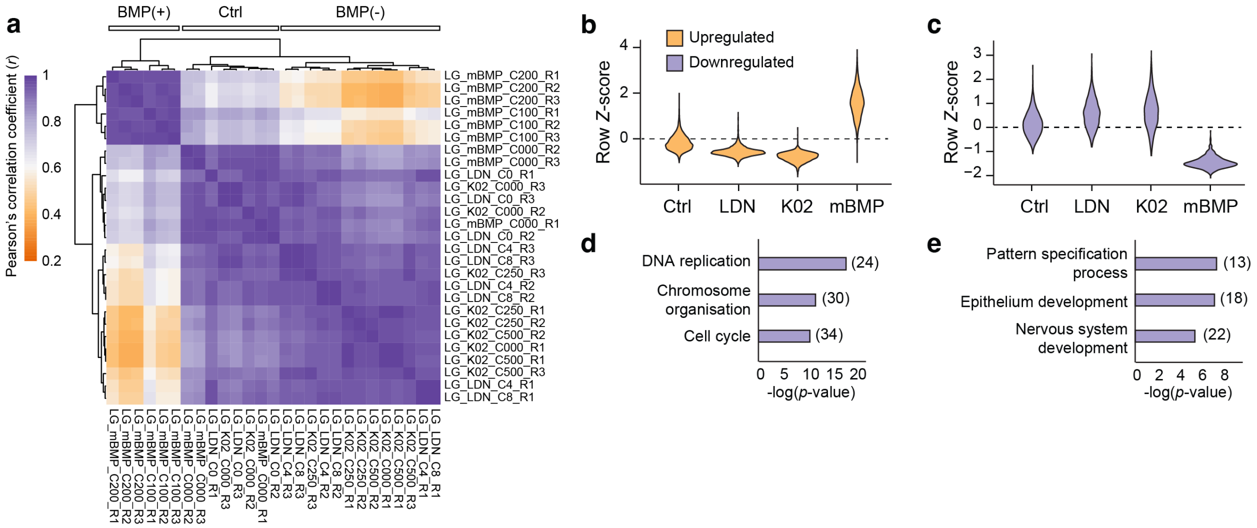

Supplementary Fig. 25 | Functional transcriptomics of BMP signal manipulation experiments. a, Pearson's correlation analysis based on 881 differentially expressed genes (fold-change > 4, $p < 0.001$) under the manipulation of BMP signals at the late gastrula stage. Each row and column represents one sample. Pearson's correlation coefficient (r) measures the similarity of samples' transcriptomes: purple being the highest similarity and orange the lowest. **b** and **c**, Violin plots showing the expression distribution of BMP-upregulated (orange) (**b**) and BMP-downregulated (purple) (**c**) gene groups under BMP signal manipulation treatments. **d** and **e**, Gene ontology enriched biological process terms for the BMP-downregulated gene set at the late gastrula stage (**d**) and early larval stage (**e**). Numbers of genes within the functional groups are shown in parentheses.

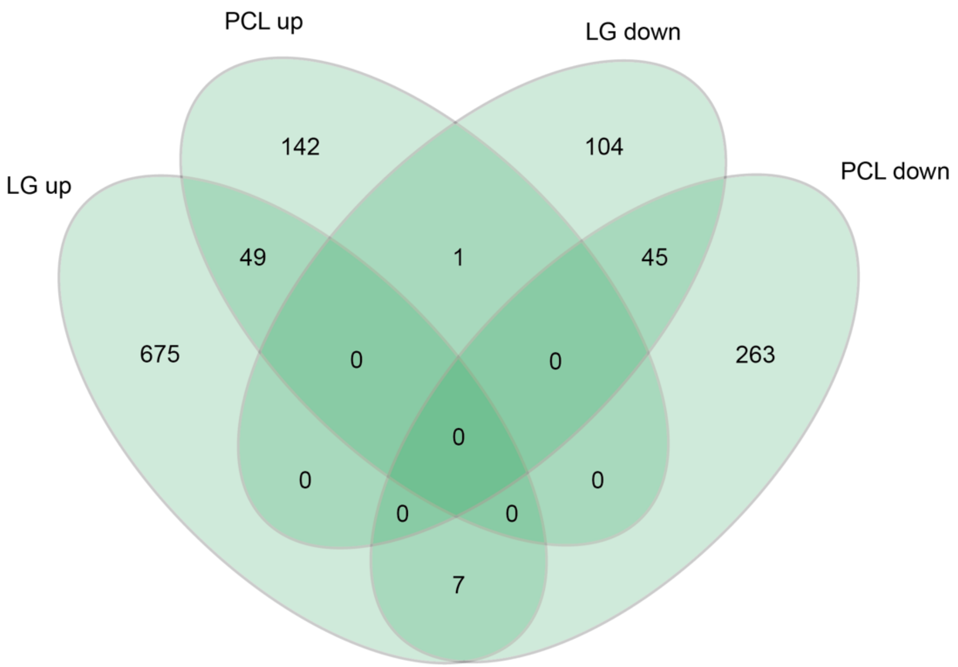

Supplementary Fig. 26 | Overlaps of differentially expressed genes. Venn diagram represents the overlaps in gene sets up- and down-regulated by BMP signalling in *L. anatina* at the late gastrula (LG) and 1-pair cirri larva (PCL) stages.

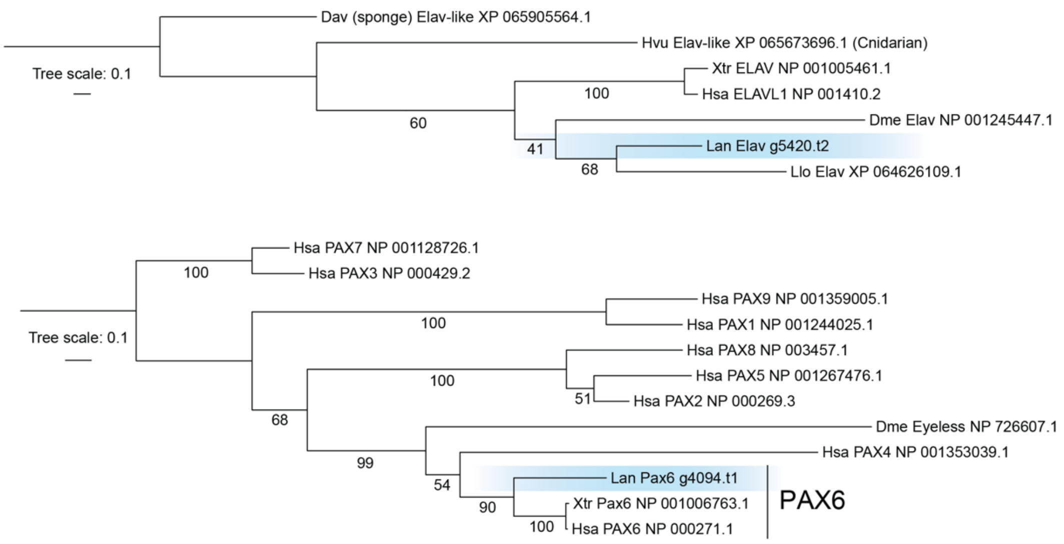

Supplementary Fig. 27 | Maximum likelihood trees of Elav and Pax6 protein sequences built using the Q.pfam+F+G4 and Q.insect+F+G4 models, respectively, in IQ-TREE. The trees support the annotation with OrthoFinder of *L. anatina* g5420 and g4094 as bona fide Elav and Pax6 proteins, respectively. In the Pax tree, all human PAX proteins (n = 9) are included as reference. Scale bar = amino acid substitutions per site. Numerical values = bootstrap support (1000 replicates). Abbreviations: Dav, *Dysidea avara* (Porifera); Dme, *Drosophila melanogaster* (Arthropoda); Hsa, *Homo sapiens* (Chordata); Hvu, *Hydra vulgaris* (Cnidaria); Lan, *Lingula anatina* (Brachiopoda); *Xenopus tropicalis* (Chordata).

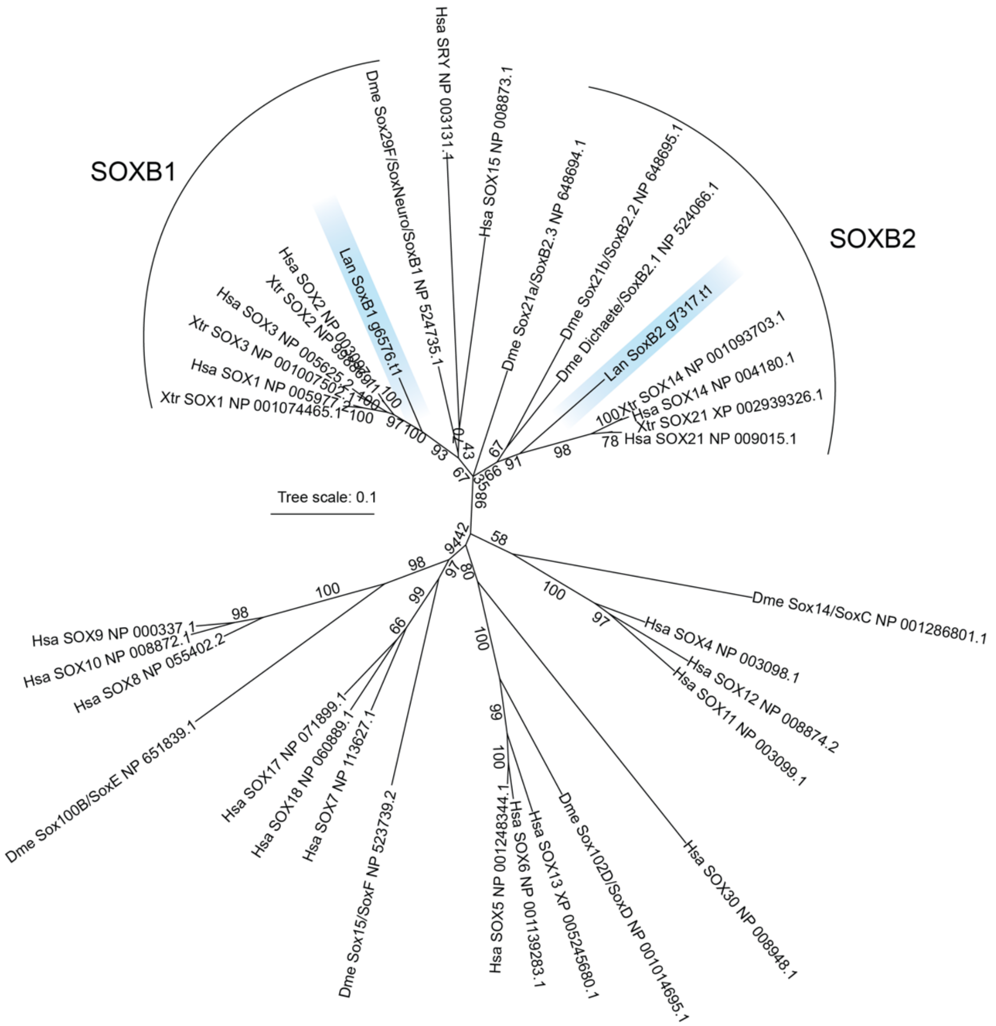

Supplementary Fig. 28 | Unrooted maximum likelihood tree of Sox sequences built using the Q.insect+F+R4 model in IQ-TREE. The tree supports the annotation of with OrthoFinder of *L. anatina* g6576 and g7317 as bona fide SoxB1 and SoxB2 proteins, respectively. All human Sox proteins (n = 20) are included as reference. Scale bar = amino acid substitutions per site. Numerical values = bootstrap support (1000 replicates). Abbreviations: Dme, *Drosophila melanogaster* (Arthropoda); Hsa, *Homo sapiens* (Chordata); Lan, *Lingula anatina* (Brachiopoda); *Xenopus tropicalis* (Chordata).

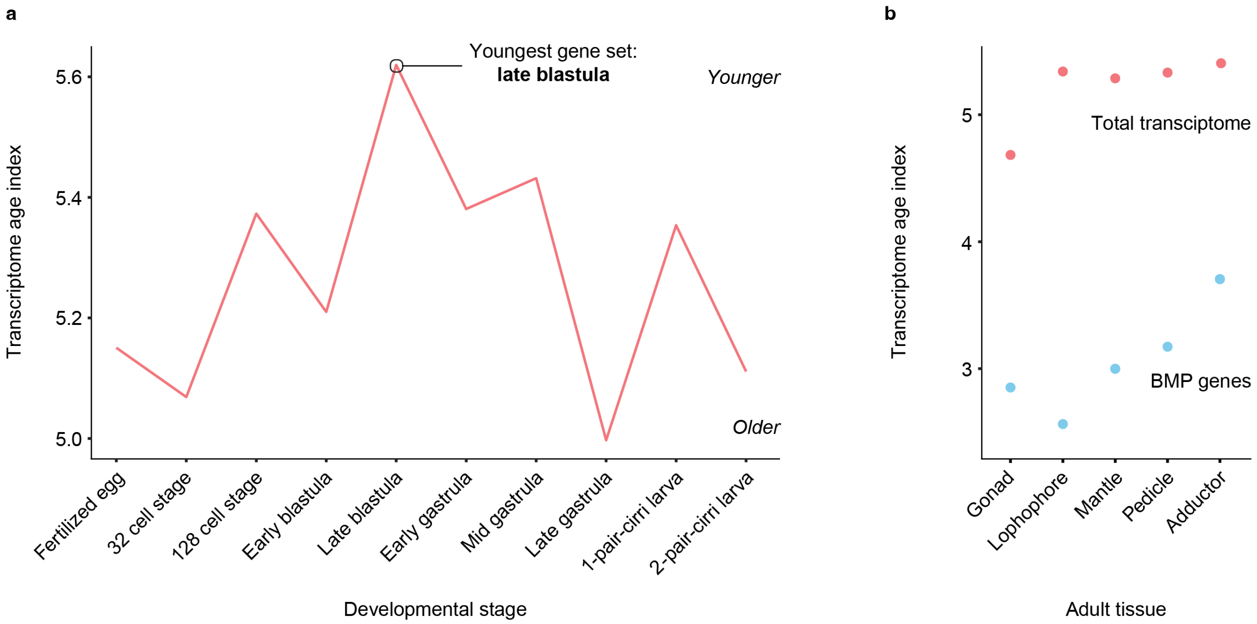

Supplementary Fig. 29 | Transcriptome age index (TAI) in *L. anatina* tissues. a, TAI of ten stages of *L. anatina* development, from the fertilised egg to the larval stage. The youngest gene set occurs at the late blastula, while the oldest is observed at the late gastrula. **b**, TAI of six adult tissues. For the total transcriptome, the oldest gene set is observed in ovary tissue and youngest in adductor muscle. The TAI of BMP pathway genes (dataset from Main Text Fig. 1c) is considerably lower than that of the overall transcriptome, reflecting their ancient evolutionary origins.

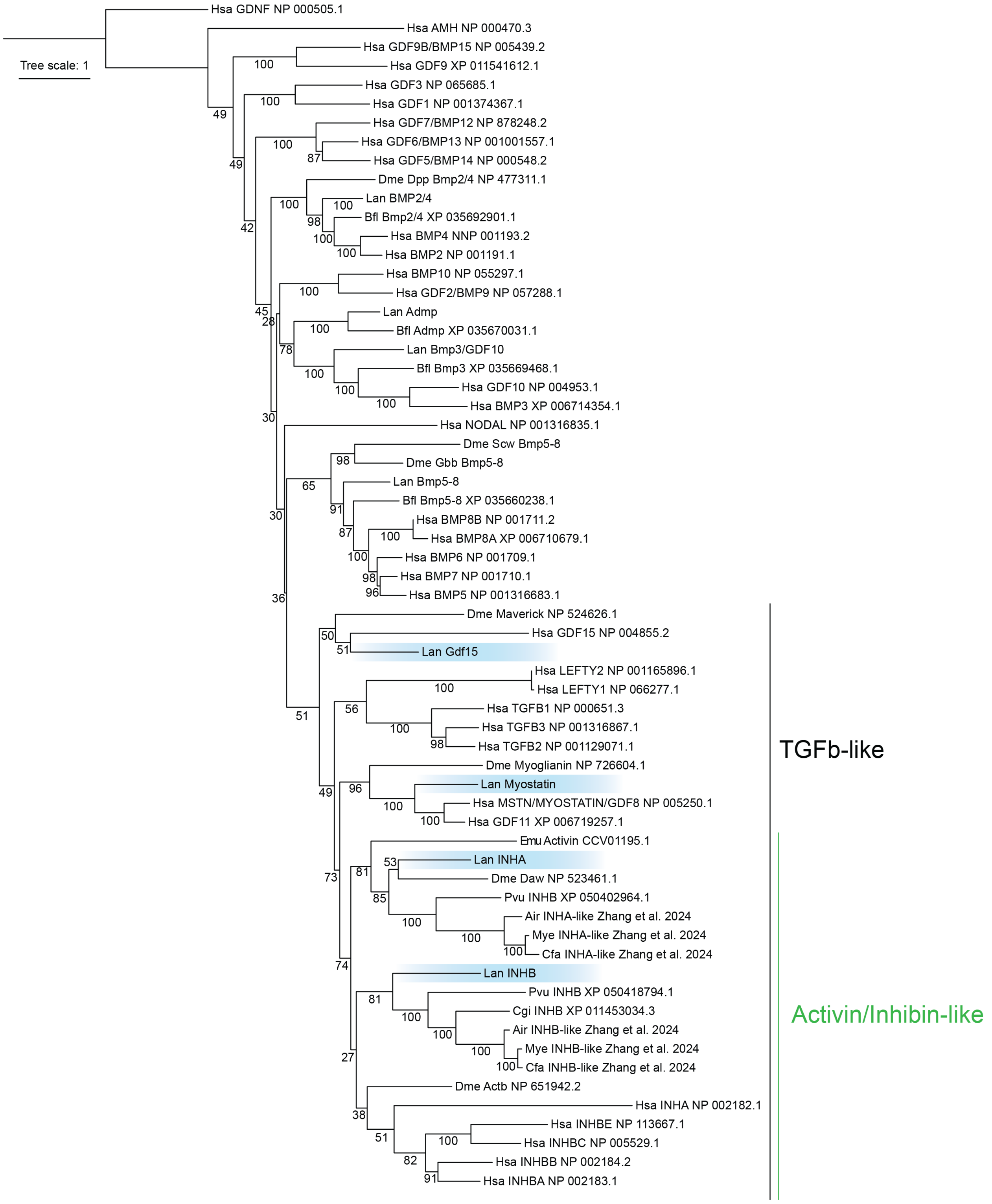

Supplementary Fig. 30 | Maximum likelihood tree TGFβ superfamily protein sequences built using the WAG+F+I+R5 model in IQ-TREE. Two activin/inhibin proteins (Inha and Inhb) are identified in *L. anatina*, alongside other TFGβ ligands Myostatin and Gdf15. Scale bar = amino acid substitutions per site. Numerical values = bootstrap support (1000 replicates).

Abbreviations: Air, *Argopecten irradians* (Mollusca); Bfl, *Branchiostoma floridae* (Chordata); Cfa, *Chlamys farreri* (Mollusca); Cgi, *Crassostrea gigas* (Mollusca); Dme, *Drosophila melanogaster* (Arthropoda); Emu*, Echinococcus multilocularis* (Platyhelminthes); Hsu, *Homo sapiens* (Chordata); Lan, *Lingula anatina* (Brachiopoda); Mye, *Mizuhopecten yessoensis* (Mollusca); Pvu, *Patella vulgata* (Mollusca).

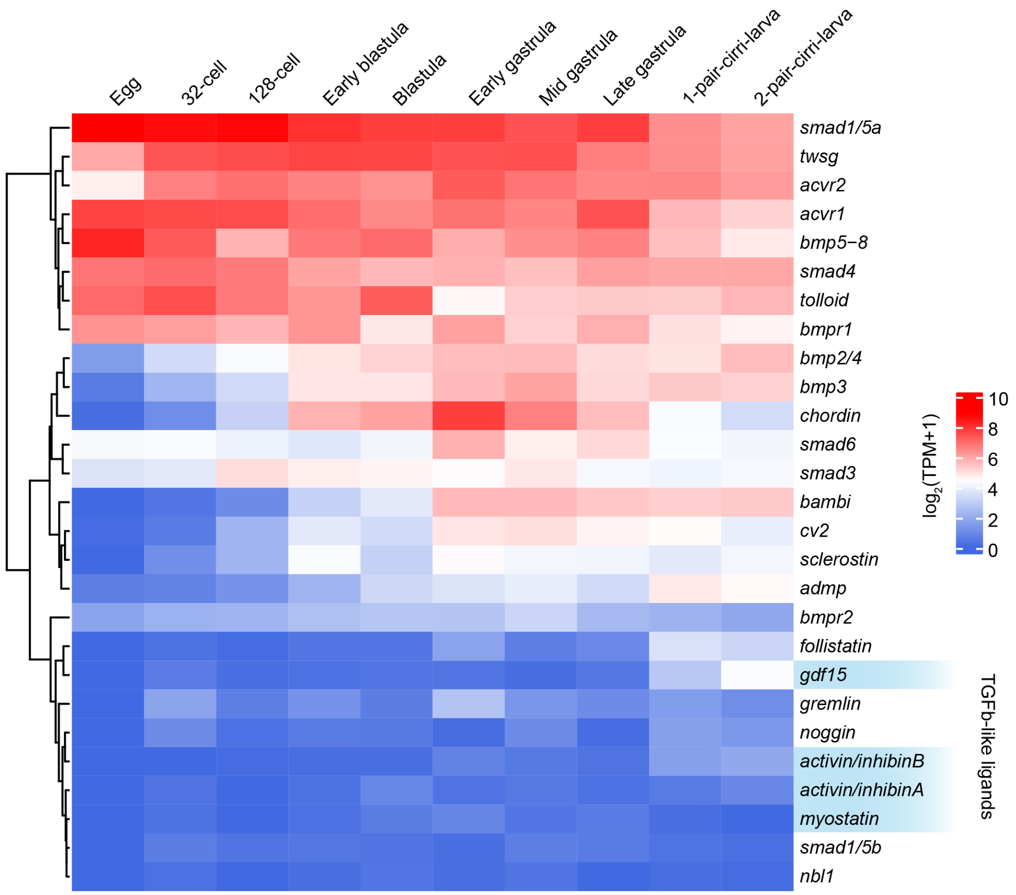

Supplementary Fig. 31 | Expression of BMP pathway genes and TGFβ ligands in *L. anatina* development. The *activin/inhibinA* and *activin/inhibinB* genes are expressed only to negligible levels. TPM, transcripts per million.

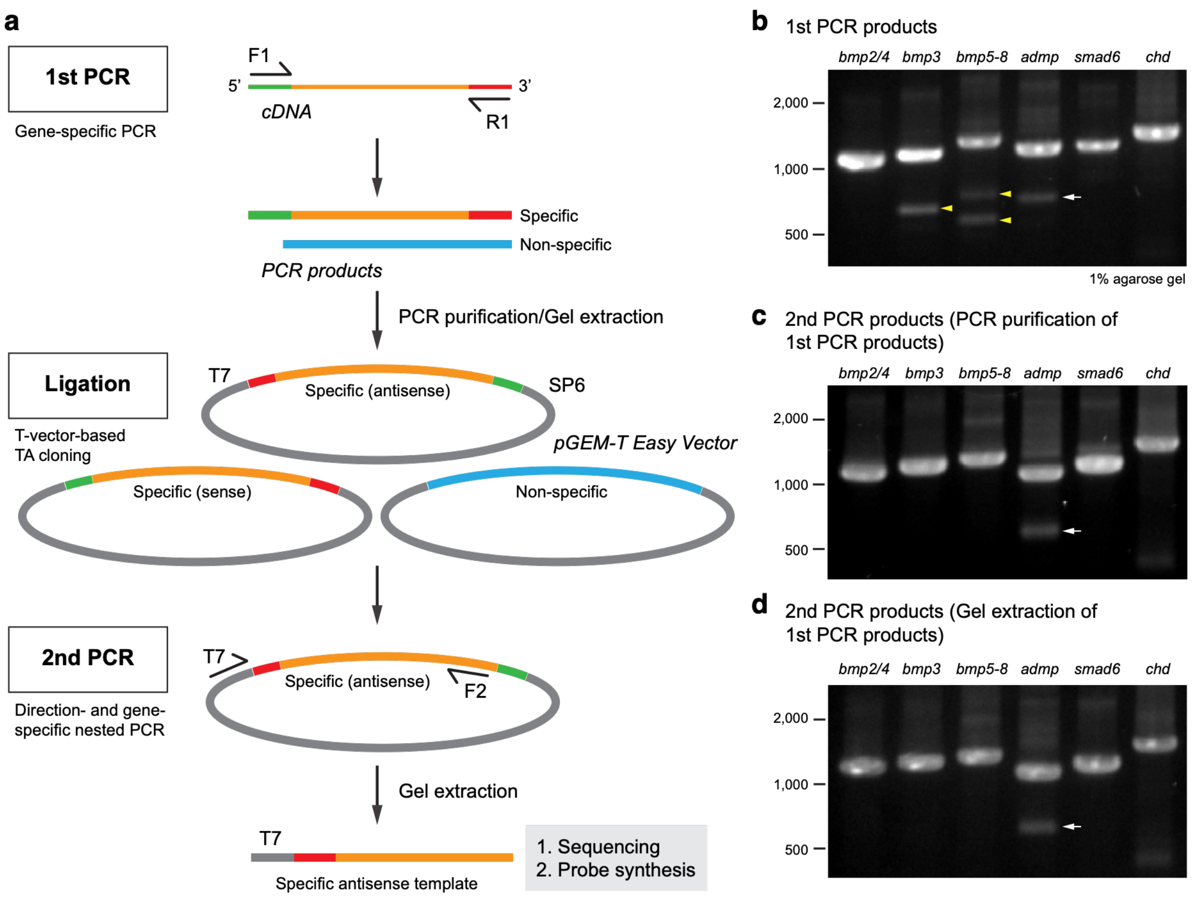

Supplementary Fig. 32 | Bacterial-cloning-free synthesis of RNA probes. a, Schematic of the process to create specific antisense DNA templates using T-vector-based ligation and nested PCR. The main products from the second PCR, which are of the predicted size, are extracted from a gel for sequencing and RNA probe synthesis. **b**, Agarose gel electrophoresis results of the first PCR products, with examples of BMP signalling components. Yellow arrowheads highlight non-specific bands. A white arrow points to a minor band of unknown origin (potentially arising from DNA secondary structures or non-specific internal insert regions). **c**, Agarose gel electrophoresis of the second PCR products, created using the first PCR products after PCR purification in the ligation reactions. **d**, Agarose gel electrophoresis of the second PCR products, obtained using ligation reactions from the first PCR products followed by gel extraction. Note that non-specific bands are eliminated after the nested (second) PCR, and no significant differences are observed between PCR purification and gel extraction when using the first PCR products for ligation.

Legends for Supplementary Data 1 to 40

Supplementary Data 1 | Sequencing statistics for the *Lingula anatina* genome.

Supplementary Data 2 | Hi-C-assisted genome scaffolding.

Supplementary Data 3 | Individual scaffold statistics for the *L. anatina* genome.

Supplementary Data 4 | Annotation of repeats in the *L. anatina* genome.

Supplementary Data 5 | Assembly statistics for the *L. anatina* genome.

Supplementary Data 6 | *L. anatina* gene annotation.

Supplementary Data 7 | Protein annotation with InterProScan.

Supplementary Data 8 | Protein annotation with KEGG orthology implemented in KofamScan.

Supplementary Data 9 | Protein annotation with eggNOG.

Supplementary Data 10 | Orthologues of *L. anatina* proteins in a mollusc (*P. vulgata*, common limpet) and a chordate (*Homo sapiens*, human) identified with OrthoFinder.

Supplementary Data 11 | Input dataset for CAFE 5 gene family evolution analysis. Abbreviations as in Supplementary Fig. 1.

Supplementary Data 12 | Genomes used for phylogenetic and comparative genomic analyses.

Supplementary Data 13 | Lophotrochozoan BMP gene repertoires. Abbreviations as in Supplementary Fig. 1.

Supplementary Data 14 | *L. anatina* BMP pathway protein sequences.

Supplementary Data 15 | Chromosome ancestral linkage group (ALG) assignments for macrosynteny analysis.

Supplementary Data 16 | Chromosome rearrangements in study species.

Supplementary Data 17 | Conserved associations of developmental genes with ALGs in *B. floridae* (Chordata), *L. anatina* (Brachiopoda), *L. longissimus* (Nemertea), *O. fusiformis* (Annelida), *P. maximus* (Mollusca) and *M. membranacea* (Bryozoa).

Supplementary Data 18 | Chi-square test for conserved ALG associations of BMP genes.

Supplementary Data 19 | Chi-square test for conserved ALG associations of Wnt genes.

Supplementary Data 20 | Expression of BMP pathway genes (TPM) during *L. anatina* embryonic development.

Supplementary Data 21 | Summary of BMP signalling manipulation experiments. Visualisation presented as main text Fig. 3a.

Supplementary Data 22 | Summary of RNA-seq samples from BMP signalling manipulation experiments.

Supplementary Data 23 | Correspondence of genome-based gene models to transcriptome.

Supplementary Data 24 | Gene expression (TPM) in BMP signalling manipulation experiments.

Supplementary Data 25 | Gene ontology (GO) analysis for genes upregulated by BMP signalling at the late gastrula stage.

Supplementary Data 26 | GO analysis for genes downregulated by BMP signalling at the late gastrula stage.

Supplementary Data 27 | GO analysis for genes upregulated by BMP signalling at the larval stage.

Supplementary Data 28 | GO analysis for genes downregulated by BMP signalling at the larval stage.

Supplementary Data 29 | Neural genes for which *in situ* hybridisation was performed.

Supplementary Data 30 | Cell cycle-related GO terms for genes downregulated by BMP signalling at the late gastrula stage. Each of the top 12 most statistically significantly enriched GO terms relates to cell proliferation, DNA replication and the cell cycle.

Supplementary Data 31 | Transcriptome age index analysis of *L. anatina* developmental stages and adult tissues.

Supplementary Data 32 | Transcriptome age index analysis of *L. anatina* late gastrula embryos and larvae under conditions of BMP signalling manipulation.

Supplementary Data 33 | Two-sided *t*-tests for differences in transcriptome age index score between BMP signalling manipulation conditions.

Supplementary Data 34 | Gene expression in *L. anatina* adult tissues.

Supplementary Data 35 | Gene expression in *L. anatina* developmental stages.

Supplementary Data 36 | Published RNA-seq datasets used to annotate the *L. anatina* genome with BRAKER.

Supplementary Data 37 | Ancestral linkage group associations of genes across bilaterians.

Supplementary Data 38 | Gene ages ('phylostrata') in the *L. anatina* genome estimated with GenEra.

Supplementary Data 39 | Supplementary genomes added to GenEra.

Supplementary Data 40 | Primers used in this work.
