# Supplementary material for: Brachiopod genome unveils the evolution of BMP signalling in bilaterian body patterning
Source: Nat Commun. 2026 Mar 12;17:3856. doi: 10.1038/s41467-026-70403-5 (PMC13121700; doi:10.1038/s41467-026-70403-5)
Supplement: Supplementary file 3 — Description of Additional Supplementary Files [file 41467_2026_70403_MOESM3_ESM.pdf]

Legends for Supplementary Data 1 to 40

Supplementary Data 1 | Sequencing statistics for the *Lingula anatina* genome.

Supplementary Data 2 | Hi-C-assisted genome scaffolding.

Supplementary Data 3 | Individual scaffold statistics for the *L. anatina* genome.

Supplementary Data 4 | Annotation of repeats in the *L. anatina* genome.

Supplementary Data 5 | Assembly statistics for the *L. anatina* genome.

Supplementary Data 6 | *L. anatina* gene annotation.

Supplementary Data 7 | Protein annotation with InterProScan.

Supplementary Data 8 | Protein annotation with KEGG orthology implemented in KofamScan.

Supplementary Data 9 | Protein annotation with eggNOG.

Supplementary Data 10 | Orthologues of *L. anatina* proteins in a mollusc (*P. vulgata*, common limpet) and a chordate (*Homo sapiens*, human) identified with OrthoFinder.

Supplementary Data 11 | Input dataset for CAFE 5 gene family evolution analysis. Abbreviations as in Supplementary Fig. 1.

Supplementary Data 12 | Genomes used for phylogenetic and comparative genomic analyses.

Supplementary Data 13 | Lophotrochozoan BMP gene repertoires. Abbreviations as in Supplementary Fig. 1.

Supplementary Data 14 | *L. anatina* BMP pathway protein sequences.

Supplementary Data 15 | Chromosome ancestral linkage group (ALG) assignments for macrosynteny analysis.

Supplementary Data 16 | Chromosome rearrangements in study species.

Supplementary Data 17 | Conserved associations of developmental genes with ALGs in *B. floridae* (Chordata), *L. anatina* (Brachiopoda), *L. longissimus* (Nemertea), *O. fusiformis* (Annelida), *P. maximus* (Mollusca) and *M. membranacea* (Bryozoa).

Supplementary Data 18 | Chi-square test for conserved ALG associations of BMP genes.

Supplementary Data 19 | Chi-square test for conserved ALG associations of Wnt genes.

Supplementary Data 20 | Expression of BMP pathway genes (TPM) during *L. anatina* embryonic development.

Supplementary Data 21 | Summary of BMP signalling manipulation experiments. Visualisation presented as main text Fig. 3a.

Supplementary Data 22 | Summary of RNA-seq samples from BMP signalling manipulation experiments.

Supplementary Data 23 | Correspondence of genome-based gene models to transcriptome.

Supplementary Data 24 | Gene expression (TPM) in BMP signalling manipulation experiments.

Supplementary Data 25 | Gene ontology (GO) analysis for genes upregulated by BMP signalling at the late gastrula stage.

Supplementary Data 26 | GO analysis for genes downregulated by BMP signalling at the late gastrula stage.

Supplementary Data 27 | GO analysis for genes upregulated by BMP signalling at the larval stage.

Supplementary Data 28 | GO analysis for genes downregulated by BMP signalling at the larval stage.

Supplementary Data 29 | Neural genes for which *in situ* hybridisation was performed.

Supplementary Data 30 | Cell cycle-related GO terms for genes downregulated by BMP signalling at the late gastrula stage. Each of the top 12 most statistically significantly enriched GO terms relates to cell proliferation, DNA replication and the cell cycle.

Supplementary Data 31 | Transcriptome age index analysis of *L. anatina* developmental stages and adult tissues.

Supplementary Data 32 | Transcriptome age index analysis of *L. anatina* late gastrula embryos and larvae under conditions of BMP signalling manipulation.

Supplementary Data 33 | Two-sided *t*-tests for differences in transcriptome age index score between BMP signalling manipulation conditions.

Supplementary Data 34 | Gene expression in *L. anatina* adult tissues.

Supplementary Data 35 | Gene expression in *L. anatina* developmental stages.

Supplementary Data 36 | Published RNA-seq datasets used to annotate the *L. anatina* genome with BRAKER.

Supplementary Data 37 | Ancestral linkage group associations of genes across bilaterians.

Supplementary Data 38 | Gene ages (‘phylostrata’) in the *L. anatina* genome estimated with GenEra.

Supplementary Data 39 | Supplementary genomes added to GenEra.

Supplementary Data 40 | Primers used in this work.
