# Supplementary material for: Brachiopod genome unveils the evolution of BMP signalling in bilaterian body patterning
Source: Nat Commun. 2026 Mar 12;17:3856. doi: 10.1038/s41467-026-70403-5 (PMC13121700; doi:10.1038/s41467-026-70403-5)
Supplement: Supplementary file 5 — Reporting Summary [file 41467_2026_70403_MOESM5_ESM.pdf]

nature portfolio

Corresponding author(s): Yi-Jyun Luo

Last updated by author(s): Feb 10, 2026

Reporting Summary

Nature Portfolio wishes to improve the reproducibility of the work that we publish. This form provides structure for consistency and transparency in reporting. For further information on Nature Portfolio policies, see our Editorial Policies and the Editorial Policy Checklist.

Statistics

For all statistical analyses, confirm that the following items are present in the figure legend, table legend, main text, or Methods section.

n/a	Confirmed	
☐	☒	The exact sample size (*n*) for each experimental group/condition, given as a discrete number and unit of measurement
☐	☒	A statement on whether measurements were taken from distinct samples or whether the same sample was measured repeatedly
☐	☒	The statistical test(s) used AND whether they are one- or two-sided<br>*Only common tests should be described solely by name; describe more complex techniques in the Methods section.*
☒	☐	A description of all covariates tested
☐	☒	A description of any assumptions or corrections, such as tests of normality and adjustment for multiple comparisons
☐	☒	A full description of the statistical parameters including central tendency (e.g. means) or other basic estimates (e.g. regression coefficient) AND variation (e.g. standard deviation) or associated estimates of uncertainty (e.g. confidence intervals)
☐	☒	For null hypothesis testing, the test statistic (e.g. *F*, *t*, *r*) with confidence intervals, effect sizes, degrees of freedom and *P* value noted<br>*Give P values as exact values whenever suitable.*
☒	☐	For Bayesian analysis, information on the choice of priors and Markov chain Monte Carlo settings
☒	☐	For hierarchical and complex designs, identification of the appropriate level for tests and full reporting of outcomes
☒	☐	Estimates of effect sizes (e.g. Cohen's *d*, Pearson's *r*), indicating how they were calculated

Our web collection on statistics for biologists contains articles on many of the points above.

Software and code

Policy information about availability of computer code

Data collection	n/a
Data analysis	SyntenyFinder (https://github.com/symgenoevolab/SyntenyFinder). Code for analyses in this study is available on GitHub (https://github.com/symgenoevolab/lingula_genome) and Zenodo (https://doi.org/10.5281/zenodo.18591213).

For manuscripts utilizing custom algorithms or software that are central to the research but not yet described in published literature, software must be made available to editors and reviewers. We strongly encourage code deposition in a community repository (e.g. GitHub). See the Nature Portfolio guidelines for submitting code & software for further information.

Data

Policy information about availability of data

All manuscripts must include a data availability statement. This statement should provide the following information, where applicable:

- Accession codes, unique identifiers, or web links for publicly available datasets
- A description of any restrictions on data availability
- For clinical datasets or third party data, please ensure that the statement adheres to our policy

Genome sequencing and RNA sequencing datasets are accessible through NCBI BioProject (PRJNA1068743). The L. anatina genome is made available on NCBI with the accession GCA_051362555.1. A L. anatina genome browser with a BLAST function is available at https://marinegenomics.oist.jp/lan_3_0/viewer/info?project_id=124. The genome sequence, gene models, and functional annotations from UniProt, Pfam, InterProScan, EggNOG and KOfam are also available at this location. Genomic, transcriptomic and imaging data from this study, including the genome assembly, gene annotations, repeat annotations, gene expression

matrices and original Zeiss LSM imaging files, are available on Zenodo (https://doi.org/10.5281/zenodo.16916709). Source data are provided with this paper.

Research involving human participants, their data, or biological material

Policy information about studies with human participants or human data. See also policy information about sex, gender (identity/presentation), and sexual orientation and race, ethnicity and racism.

Reporting on sex and gender	n/a
Reporting on race, ethnicity, or other socially relevant groupings	n/a
Population characteristics	n/a
Recruitment	n/a
Ethics oversight	n/a

Note that full information on the approval of the study protocol must also be provided in the manuscript.

Field-specific reporting

Please select the one below that is the best fit for your research. If you are not sure, read the appropriate sections before making your selection.

[x] Life sciences [ ] Behavioural & social sciences [ ] Ecological, evolutionary & environmental sciences

For a reference copy of the document with all sections, see nature.com/documents/nr-reporting-summary-flat.pdf

Life sciences study design

All studies must disclose on these points even when the disclosure is negative.

Sample size	n/a
Data exclusions	n/a
Replication	For RNA sequencing, three independent batches of embryos were used as biological replicates. For in situ hybridization, 20–50 embryos were analysed for each staining.
Randomization	n/a
Blinding	n/a

Reporting for specific materials, systems and methods

We require information from authors about some types of materials, experimental systems and methods used in many studies. Here, indicate whether each material, system or method listed is relevant to your study. If you are not sure if a list item applies to your research, read the appropriate section before selecting a response.

Materials & experimental systems

n/a	Involved in the study	
[ ]	[x]	Antibodies
[x]	[ ]	Eukaryotic cell lines
[x]	[ ]	Palaeontology and archaeology
[ ]	[x]	Animals and other organisms
[x]	[ ]	Clinical data
[x]	[ ]	Dual use research of concern
[x]	[ ]	Plants

Methods

n/a	Involved in the study	
[x]	[ ]	ChIP-seq
[x]	[ ]	Flow cytometry
[x]	[ ]	MRI-based neuroimaging

Antibodies

Antibodies used	Rabbit anti-phospho-Smad1/5 antibody (1:200; Cell signalling 9511S), Rabbit anti-phospho-Histone H3 (Ser10) antibody (1:100; Millipore 06-570).

Validation	The commercial anti-phospho-Smad1/5 antibody was raised against a phosphopeptide antigen that shares the same epitope in humans and the brachiopod Lingula anatina, and has been validated in other marine invertebrates including amphioxus, sea urchins and hemichordates. The commercial anti-phospho-Histone H3 antibody targets a conserved antigen found across metazoans.

Animals and other research organisms

Policy information about [studies involving animals](); [ARRIVE guidelines]() recommended for reporting animal research, and [Sex and Gender in Research]()

Laboratory animals	n/a
Wild animals	Between 2013 to 2016, gravid adults of the brachiopod L. anatina (approximately 150 to 200) were collected each August during low tide from Kasari Bay, Amami Island, Japan (28.440583 N, 129.667608 E).
Reporting on sex	n/a
Field-collected samples	Around 50 individuals were housed in a 10 L aerated seawater tank and fed daily with 25 mL/tank of Chaetoceros calcitrans (5×107 cells/mL).
Ethics oversight	n/a

Note that full information on the approval of the study protocol must also be provided in the manuscript.

Plants

Seed stocks	n/a
Novel plant genotypes	n/a
Authentication	n/a
